# Identification of Drug-Induced Multichannel Block and Proarrhythmic Risk in Humans Using Continuous T Vector Velocity Effect Profiles Derived From Surface Electrocardiograms

**DOI:** 10.3389/fphys.2020.567383

**Published:** 2020-09-18

**Authors:** Werner Bystricky, Christoph Maier, Gary Gintant, Dennis Bergau, David Carter

**Affiliations:** ^1^Clinical Pharmacology and Pharmacometrics, AbbVie, Inc., North Chicago, IL, United States; ^2^Department of Medical Informatics, Heilbronn University, Heilbronn, Germany; ^3^Integrated Sciences and Technology, AbbVie, Inc., North Chicago, IL, United States

**Keywords:** cardiac safety, TVV, CiPA, QT prolongation, J-Tpeak interval, ion channel block, ventricular repolarization

## Abstract

We present continuous T vector velocity (TVV) effect profiles as a new method for identifying drug effects on cardiac ventricular repolarization. TVV measures the temporal change in the myocardial action potential distribution during repolarization. The T vector dynamics were measured as the time required to reach p percent of the total T vector trajectory length, denoted as Tr(p), with p in {1, …, 100%}. The Tr(p) values were individually corrected for heart rate at each trajectory length percentage p. Drug effects were measured by evaluating the placebo corrected changes from baseline of Tr(p)c jointly for all p using functional mixed effects models. The p-dependent model parameters were implemented as cubic splines, providing continuous drug effect profiles along the entire ventricular repolarization process. The effect profile distributions were approximated by bootstrap simulations. We applied this TVV-based analysis approach to ECGs available from three published studies that were conducted in the CiPA context. These studies assessed the effect of 10 drugs and drug combinations with different ion channel blocking properties on myocardial repolarization in a total of 104 healthy volunteers. TVV analysis revealed that blockade of outward potassium currents alone presents an effect profile signature of continuous accumulation of delay throughout the entire repolarization interval. In contrast, block of inward sodium or calcium currents involves acceleration, which accumulates during early repolarization. The balance of blocking inward versus outward currents was reflected in the percentage p_zero_ of the T vector trajectory length where accelerated repolarization transitioned to delayed repolarization. Binary classification using a threshold p_zero_ = 43% separated predominant hERG channel blocking drugs with potentially higher proarrhythmic risk (moxifloxacin, dofetilide, quinidine, chloroquine) from multichannel blocking drugs with low proarrhythmic risk (ranolazine, verapamil, lopinavir/ritonavir) with sensitivity 0.99 and specificity 0.97. The TVV-based effect profile provides a detailed view of drug effects throughout the entire ventricular repolarization interval. It enables the evaluation of drug-induced blocks of multiple cardiac repolarization currents from clinical ECGs. The proposed p_zero_ parameter enhances identification of the proarrhythmic risk of a drug beyond QT prolongation, and therefore constitutes an important tool for cardiac arrhythmia risk assessment.

## Introduction

The implementation of the ICH E14 (ICH, 2005a) and S7B (ICH, 2005b) guidelines in 2005 represented a turning point in regulatory practices for drug approval. Their release was in response to cases of sudden cardiac death reported in the preceding decade, which resulted in withdrawal of several drugs from the market. The guidelines were inspired by the insight that a block of the hERG/IKr ion current delays ventricular repolarization and creates an electrophysiological environment that favors the development of the rare ventricular arrhythmia torsade de pointes (TdP) (Haverkamp et al., 2000). In the surface ECG, the most obvious effect of hERG/IKr-block is delayed repolarization manifested as prolongation of the QTc interval. Consequently, clinical guidance set the focus on this feature, and up to the present, careful monitoring of the QT interval remains an essential part in the drug approval process.

This change in regulatory practices was highly effective in preventing the approval of new drugs with unexpected cardiotoxic effects (Sager et al., 2014). However, QT prolongation turned out to be a relatively non-specific predictor of TdP proarrhythmia (Hondeghem, 2008) and could potentially terminate the development of promising compounds unnecessarily (Roden, 2004). Further research has revealed that simultaneous block of other ion channels, especially the late sodium and the L-type calcium channel, can attenuate the proarrhythmic effects of pure hERG/IKr block despite the presence of QT-prolongation (Redfern et al., 2003; Martin et al., 2004).

Recognizing the role of multiple ion currents in defining drug effects on repolarization, the comprehensive *in vitro* proarrhythmia assay (CiPA) initiative was launched in 2013 (Sager et al., 2014) with the mission to “engineer an assay for assessment of the proarrhythmic potential of new drugs that has improved specificity compared with the hERG assay plus Thorough QT study” (CiPA, 2020). The CiPA initiative is comprised of four components (Gintant et al., 2016), three of which are preclinical tests: (1) voltage-clamp based assessment of blocks in seven cardiac ion channels, (2) an *in silico* modeling approach based on modifications (Dutta et al., 2017) of the O’Hara-Rudy ventricular myocyte model (O’Hara et al., 2011) to simulate the effects of expected ion channel blocks onto the action potential on a cellular level, and (3) the use of human induced pluripotent stem cell-derived cardiomyocytes to confirm predicted effects *in vitro.*

For a list of 28 drugs, the proarrhythmic potential was ranked (high – intermediate – low) according to known properties (Gintant et al., 2016), and their ion-channel effects were thoroughly characterized (Crumb et al., 2016). The qNet metric (Dutta et al., 2017) was suggested as a surrogate for proarrhythmia, based on quantification of the balance of charge transport over four essential inward and outward currents, as obtained from the cellular action potential (AP) simulation. The qNet metric demonstrated excellent performance for separating the set of CiPA drugs into the risk categories (Li et al., 2019).

Besides the CiPA context, an advantage of human *in silico* drug trials over animal studies in predicting cardiotoxicity of 62 compounds was demonstrated (Passini et al., 2017). Simpler approaches compared to qNet suggested to only consider the net difference in block between depolarizing and repolarizing currents (Bnet) (Mistry, 2018; Han et al., 2019). More complex approaches extend the cellular simulation to multiple scales up to the level of the whole heart in three dimensions (Zemzemi et al., 2013; Okada et al., 2015; Hwang et al., 2019; Yang et al., 2020) and combine simulation with machine learning techniques to assess the arrhythmogenic risk of drugs (Sahli-Costabal et al., 2020). Others start directly from a chemical drug representation to predict potential cardiotoxic effects, e.g., by means of deep artificial neural networks (Cai et al., 2019).

Despite the impressive findings obtained from *in silico* and *in vitro* models, there are discrepancies between model predictions and experimental observations, in particular for multichannel blocking drugs (Britton et al., 2017). For *in vitro* models, results depend on the experimental protocol, and quality standards as well as experimental conditions need to be taken into account (Ridder et al., 2020). Consequently, the fourth CiPA component stipulates the complementary use of early, intensive clinical ECG monitoring to assess cardiac effects in humans, to confirm model predictions and to identify potential unanticipated threats.

In this context, research continues to find more specific clinical ECG biomarkers of a drug’s proarrhythmic risk (Vicente et al., 2015). Most approaches focus on the ST-T region of the ECG as it corresponds to cardiac repolarization. A principal distinction is possible between methods targeting representative beat characteristics versus dynamic beat-to-beat aspects of repolarization. Beat-to-beat QT-interval variability was introduced as a marker of temporal lability of repolarization linked to arrhythmic susceptibility (Berger et al., 1997), and various algorithms and markers exist (Baumert et al., 2016). Alternative approaches assess QT interval dynamics by grouping QT according to heart rate (Fossa and Zhou, 2010), and suggest characterization of electrocardiographic restitution by quantifying QT-TQ-interval relationship (Fossa, 2017). Finally, T-wave alternans analysis represents an important dynamic technique quantifying the occurrence of a specific type of modulation of T-wave morphology or amplitude. Its electrophysiological basis is well-understood (Verrier et al., 2011), and the phenomenon is causally linked to arrhythmogenic risk (Verrier and Malik, 2013). However, application of these dynamic techniques requires ECG data sequences of sufficient length. Moreover, their utilization has largely concentrated on arrhythmic risk stratification of patients under well-defined disease conditions. Comprehensive studies exploring their performance in pro-arrhythmic risk assessment of drugs are still missing.

In the group of approaches addressing static properties of repolarization, candidate features (Brennan and Tarassenko, 2012) have included morphological ECG properties like notching, flattening or asymmetry of the T-wave (Graff et al., 2009), as well as vectorcardiographic biomarkers like QRS-T angle (Acar et al., 1999), and early and late repolarization duration (Couderc, 2009). In a comprehensive series of three CiPA ECG studies (Johannesen et al., 2014; Johannesen et al., 2016; Vicente et al., 2019), the J-T_peak_c interval was identified as the most suitable biomarker to separate multi-channel blocking drugs with low proarrhythmic risk from predominant hERG blockers with high proarrhythmic risk (sensitivity 0.82, specificity 0.77) (Vicente et al., 2016). Approaches to identify the T-wave peak in a more reproducible and stable way have been suggested (Hnatkova et al., 2019). Recently, we presented a TVV-based analysis approach as a new method for electrocardiographic repolarization assessment and demonstrated that it outperforms J-T_peak_c as a biomarker for identification of multi-channel blocking drugs (Bystricky et al., 2019).

The rationale for this TVV approach may be outlined as follows: the T vector at a certain point in time during repolarization reflects the spatial gradients of the action potentials distributed over the entire myocardium as recorded from the body surface ECG. Any change of the T vector with time reflects a change of the myocardial potential distribution. Thus, the T vector trajectory between the J-point (the end of depolarization) and the end of the T wave is a three-dimensional descriptor of the state transition of the myocardial action potentials during repolarization. TVV denotes the velocity at which the T vector moves along the T vector trajectory, thus representing myocardial potential dynamics. The action potential dynamics of a single myocardial cell are determined by complex interactions of currents flowing across multiple ion channels that can be described in terms of a set of mathematical differential equations (O’Hara et al., 2011; Dutta et al., 2017; Tomek et al., 2019). Drugs affecting multiple ion channels will impact the repolarization dynamics at different times during the action potential. We hypothesize that drug-induced changes throughout the time course of repolarization will be reflected in the TVV along the T vector trajectory.

The purpose of our current study is to present an extension of the TVV methodology which allows for continuous quantification of changes in the temporal dynamics over the entire phase of repolarization. We apply this method to the published data from all three CiPA ECG studies in order to provide reference results for these publicly available data sets and corroborate our suggested interpretation with additional support. Finally, we illustrate the potential of TVV analysis by showing that a scalar biomarker p_zero_, extracted from the TVV drug effect profile, can separate predominant hERG channel blocking drugs with potentially higher proarrhythmic risk from multichannel blocking drugs with low proarrhythmic risk with excellent performance.

## Materials and Methods

### Study Data

We used de-identified data from the three CiPA ECG studies that are publicly available from PhysioNet (Goldberger et al., 2000).

The first study (Study A, ECGRDVQ) (Johannesen et al., 2014) was a randomized, double-blind, 5-period crossover clinical trial with 22 healthy subjects. Its aim was to investigate whether multichannel blocking drugs with different potentials for blocking potassium, late sodium, and calcium currents can be differentiated by their effect on the ECG. In the morning of each 24 h treatment period, all subjects received a single dose of one of the drugs dofetilide (500 μg), quinidine sulfate (400 mg), ranolazine (1,500 mg), verapamil hydrochloride (120 mg), or placebo. Triplicate 10-s resting ECGs were recorded, and serum PK samples were taken at 16 pre-defined time-points (pre-dose and 0.5, 1, 1.5, 2, 2.5, 3, 3.5, 4, 5, 6, 7, 8, 12, 14, 24 h post-dose). The washout period between treatments was 7 days (for further details see, Johannesen et al., 2014).

The second study (Study B, ECGDMMLD) (Johannesen et al., 2016) was a randomized, double-blinded, 5-period crossover clinical trial in 22 healthy subjects. It addressed the electrophysiological responses to hERG/IKr current blocking drugs with and without the addition of blockade of either late sodium or L-type calcium current blocking drugs. The 5 treatment periods included dofetilide alone, mexiletine without and with dofetilide, lidocaine without and with dofetilide, moxifloxacin without and with diltiazem, and placebo. In each period, subjects were dosed three times per day, in the morning (hour 0), afternoon (hour 4) and evening (hour 9.5) as described in Table 1. Serum PK samples were taken after each dosing, and triplicate 10-s resting ECGs were recorded at four timepoints at 30 min intervals after each dosing event. For details see (Johannesen et al., 2016).

**TABLE 1:** Dosing schema for study B, modified from Johannesen et al. (2016).

Treatment period	Morning dose	Afternoon dose	Evening dose
Placebo (Pla)	Pla	Pla	Pla
Dofetilide (Dof)	Pla	Dof	Dof
Mexiletine (Mex) + Dofetilide	Mex	Mex + Dof	Mex + Dof
Lidocaine (Lid) + Dofetilide	Lid	Lid + Dof	Lid + Dof
Moxifloxacin (Mox) + Diltiazem (Dil)	Mox	Mox	Mox + Dil

The third study (Study C, CiPA) (Vicente et al., 2019) was designed as a prospective, small sample size CiPA validation study to assess the effect of multi-ion channel-blocking drugs on ECG parameters. It consisted of two parts:

• Part 1 was a double-blind, randomized, placebo-controlled, one period, parallel designed study to assess the effect of three balanced blockers (ranolazine, verapamil, and lopinavir + ritonavir), one predominant hERG blocker (chloroquine), and one placebo on the QTc and J-T_peak_c intervals in 50 healthy subjects. The four drugs and placebo were administered to 10 subjects in one period of three consecutive days to achieve low and high drug exposure.
• Part 2 was a double-blind, randomized, two-period crossover designed study to assess the effect of hERG block (dofetilide) versus calcium block (diltiazem) on the QTc and J-T_peak_c intervals in 10 healthy subjects. In the dofetilide alone period, subjects received dofetilide on days 1 and 3. In the diltiazem + dofetilide period, subjects received diltiazem alone on days 1 and 2, and diltiazem + dofetilide on day 3.

In both parts, triplicate ECGs and PK samples were taken at multiple timepoints during the 3 days.

Details about the time courses of the drug plasma concentrations during the treatment phases of all three studies are given in the Supplementary File S2.

### ECG Processing

All ECG files were analyzed with eECG/ABBIOS (AbbVie, Inc.’s proprietary, validated, ECG analysis system) in a semi-automated manner. ECGs were reviewed to identify artifacts, abnormal heartbeats, and unreliable automated annotations. All normal beats with acceptable annotations were used in the analysis. ECGs of concern were manually reviewed to identify and annotate a minimum of 3 heartbeats per ECG with T-waves unaffected by artifacts, and consistently placed T annotations. To ensure consistent placement of T annotations, reviewing was performed unblinded with respect to the subject and timepoint. 6 ECGs out of 5232 in Study A, 3 ECGs out of 4211 in Study B, and 0 ECG out of 5749 in Study C were not included in the analysis due to bad signal quality.

For each ECG, the 12 lead signals were adjusted to the isoelectric lines (defined by the median amplitude of the PQ interval and interpolated between the heart beats by a cubic spline), low-pass filtered (bidirectional Bessel filter with 36 Hz), and exported as an annotated ECG file in HL7 format (aECG) with 500 Hz sampling frequency, including the P, Q, J, and Tend annotations. The aECG files were loaded into, and further processed using the R (RCoreTeam, 2020) and the Julia (Bezanson et al., 2020) systems.

The time course of the three-dimensional T vector in a beat was calculated by means of the inverse Dower transformation (Dower et al., 1980).

### Derivation of T Vector Trajectory Duration Curves

The T vector dynamics was measured for each normal heartbeat as the time required to reach p percent of the total T vector trajectory length, denoted as Tr(p) and referred to in our manuscript as trajectory (time) quantile p, with p in {1, 2, …, 100%}.

In order to exclude possible late depolarization effects, the beginning of the T vector trajectory was determined as the J-point plus 20 ms. The T vector trajectory was determined as described in Bystricky et al. (2019). The trajectory quantiles for an ECG were, individually for each p, calculated as average of the beat related Tr(p) values.

**FIGURE 1:**
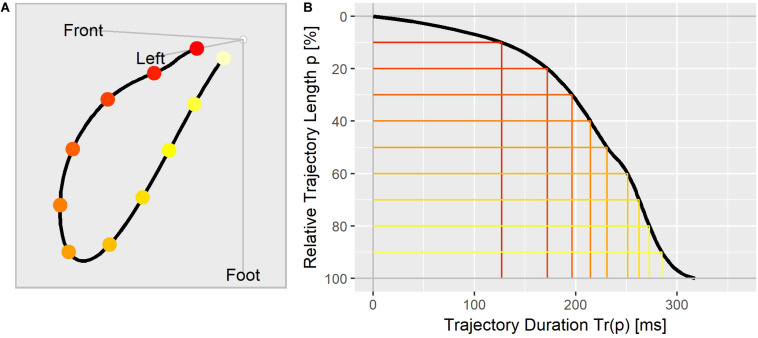
**(A)** The T vector trajectory displayed in the body’s physical orientation, divided into 10 equally long segments and colored from red (J-point) to yellow (T_end_). **(B)** T vector trajectory duration curve indicating the elapsed time Tr(p) (x-axis) at which the corresponding percentage of the trajectory length (y-axis) is reached. The colored lines correspond to the trajectory points as marked in **(A)**.

To visualize the repolarization dynamic of an ECG, we aligned the Tr(p) values in a trajectory duration curve (Figure 1B), where the relative distance covered along the T vector trajectory (Figure 1A) is displayed on the y axis (in % from top to bottom) and the corresponding duration in milliseconds is shown along the x axis.

Assuming a power law dependency of Tr(p) from the heart rate of the form *Tr*(*p*)∼β_*p*_*RR*^α_*p*_^ with RR as the average beat interval of an ECG in seconds, we determined the heart rate correction exponents α_*p*_ for each trajectory length percentage p by fitting a linear mixed effects model of the form

$$\log\left(Tr\left(p\right)\right)=\log\left(\beta_{p}\right)+\alpha_{p}\log\left(RR\right)+\varepsilon$$

to all drug free data of studies A, B, and C with subject as random effect for the intercept *log*⁡(β_*p*_) and slope α_*p*_ (R method lme4::lmer). The heart rate corrected trajectory quantiles for an ECG were calculated as

$$Tr\left(p\right)c=Tr\left(p\right)/RR^{\alpha_{p}}$$

We determined the average trajectory duration curve under placebo condition in study A by fitting the mixed effects model

$$Tr\left(p\right)c_{i}=\theta_{0}\left(p\right)+\eta_{i}\left(p\right)$$

for a given trajectory length percentage p with θ_0_(*p*) as fixed effect and η_*i*_(*p*) as random effect for subject i. To visualize drug induced changes to the trajectory duration curve, we modeled the trajectory quantiles under quinidine treatment in study A for the individual trajectory length percentages p by the following mixed effects exposure response model:

$$Tr\left(p\right)c_{i,k}=\left(\theta_{0}\left(p\right)+\eta_{0,i}\left(p\right)\right)+\left(\theta_{1}\left(p\right)+\eta_{1,i}\left(p\right)\right)\times C_{i,k}$$

*C*_*i,k*_ is the drug concentration for subject i at time k; θ_0_(*p*) and θ_1_(*p*) are the fixed effects; η_0,*i*_(*p*), η_1,*i*_(*p*) are the random effects for subject i. The trajectory duration curve under quinidine was calculated as the model predictions for the individual *p*-values at the c_max_ quinidine concentration.

### Calculation of Drug Effects on Tr(p)c

The drug effects on the individual Tr(p)c parameters were assessed by modeling the placebo corrected change from baseline, where Tr(p)c was calculated as the average value from the replicate ECGs for the given subject and timepoint. Given the different designs in studies A, B, and C, the following mixed effects models were used.

### Study A

We used a mixed effects exposure response model for describing the placebo corrected change from baseline of Tr(p)c with linear dependency on the drug concentration:

$$\Delta \Delta Tr\left(p\right)c_{ik}=\left(\theta_{0}\left(p\right)+\eta_{0,i}\left(p\right)\right)+\left(\theta_{1}\left(p\right)+\eta_{1,i}\left(p\right)\right)\times C_{ik}$$

ΔΔ*Tr*(*p*)*c*_*ik*_ is the placebo corrected change from baseline of Tr(p)c for subject i at time k; θ_0_(*p*) and _θ_1_ (p)_ are the fixed effect parameters for intercept and slope; η_0,*i*_(*p*) and η_1,*i*_(*p*) are the subject specific random effect parameters for intercept and slope; *C*_*ik*_ is the drug concentration for subject i at time k.

### Study B

We measured the drug effects for the morning, afternoon, and evening dosing phases as the average of the placebo corrected change from baseline Tr(p)c values using the mixed effects model:

$$\Delta \Delta Tr\left(p\right)c_{ik}=\theta \left(p\right)+\eta_{i}\left(p\right)$$

ΔΔ*Tr*(*p*)*c*_*ik*_ is the placebo corrected change from baseline of Tr(p)c for subject i at time k within the given dosing phase; θ(*p*) is the fixed effect parameter denoting the average drug effect; η_*i*_(*p*) is the subject specific random effect parameter.

### Study C

Given the parallel design in the study part 1, we determined the drug effect profiles by modeling the changes from baseline Tr(p)c data according to Garnett et al. (2018) using the following mixed effects exposure response approach:

$$\begin{array}{c} \Delta Tr\left(p\right)c_{ijk}=\left(\theta_{0}\left(p\right)+\eta_{0,i}\left(p\right)\right)+\theta_{1}\left(p\right)TRT_{j}\\ \;+\sum_{\iota }\left(\theta_{2,\iota }\left(p\right)+\eta_{2,i,\iota }\left(p\right)\right)\times c_{ijk,\iota }+\theta_{3}\left(p\right)NT_{k}\\ \;+\theta_{4}\left(p\right)\left(Tr\left(p\right)c_{i,j=0^{-}}\bar{Tr\left(p\right)C_{0}}\right) \end{array}$$

Δ*Tr*(*p*)*c*_*ijk*_ is the change from baseline of Tr(p)c for subject i under treatment j at time k; θ_0_(*p*) is the fixed effect population mean intercept in the absence of a treatment effect; η_0,*i*_(*p*) is the subject-specific random effect for the intercept; θ_1_(*p*) is the fixed effect associated with treatment *TRT*_*j*_ (j = 0 for placebo, j = 1 for the active drug); θ_2,ι_(*p*) and η_2,*i*,ι_(*p*) are the fixed and random effects for the slope with respect to the concentration *C*_*ijk,ι*_ of drug l (two drugs were used for the combination of dofetilide and diltiazem only); θ_3_(*p*) is the fixed effect associated with the nominal timepoint*NT*_*k*_; θ_4_(*p*) is the fixed effect associated with baseline *Tr*(*p*)*c*_*i*,*j* = 0_; $\bar{Tr\left(p\right)c_{0}}$ is the overall mean of all baseline Tr(p)c values.

The placebo corrected change from baseline effect for drug l with concentration *C*_ι_ on Tr(p)c was calculated as

$$\Delta \Delta Tr\left(p\right)c=\hat{\theta_{1}\left(p\right)}+\sum_{\iota }\hat{\theta_{2,\iota }\left(p\right)}\times C_{\iota }$$

where $\hat{\theta \ldots \left(p\right)}$ denotes the estimated parameter.

### Derivation of Continuous Drug Effect Profiles

To describe the effect of a drug continuously over the entire repolarization process, we combined the individual models for the trajectory length percentages *p* = 1, 2, …, 100 from above into one single functional mixed effects model, where the p-dependent model parameters were parametrized by polynomial splines with degree 3 (R method splines::bs). The spline’s degree of smoothing along the p range is determined by the knots, defining the spline basis functions. We used equidistantly spaced knots with boundaries *p* = 1 and *p* = 100. Since the computational effort for fitting the functional model depends on the model complexity and of the number of knots, we used for the models in study A 8 knots, 16 knots for the models in study B, and 12 knots for the models in study C. Further details about the functional modeling approach are given in the Supplementary File S3.

A continuous drug effect profile was defined as the predicted ΔΔ*Tr*(*p*)*c* for representative drug concentrations along the T vector trajectory between the length percentages *p* = 1% and *p* = 100%. It describes how a drug accelerates or delays the repolarization process by measuring the drug-induced change of the time that it takes to reach a certain percentage of the total T vector trajectory length.

We used a non-parametric two-step bootstrapping simulation approach to determine the distribution of the effect profiles with subject as primary unit for resampling. The resampled subject data were then bootstrapped with ECG as secondary unit for resampling. For a given trajectory length percentage p, the predicted ΔΔ*Tr*(*p*)*c* value was determined as the median bootstrap simulation value, and the two-sided 90% confidence band was determined as the 5 and 95% quantiles of the bootstrap simulation values. The functional models were fitted using the Julia package MixedModels (Bates et al., 2020) on a high-performance Linux cluster. Fitting the most complex functional model (combined treatment of lopinavir + ritonavir in study C with 406 model parameters and 50000 records) took approximately 1 h. Thus, we choose the number of bootstrap simulation steps as *N* = 4000 in the studies A and B, and 1000 in study C.

### Determination of p_zero_

Typically for multi-channel blocking drugs, the effect profiles are negative in the early repolarization phase (indicating accelerated repolarization) and positive in the late repolarization phase (indicating delayed repolarization). In order to describe the effective degree of balance of multiple channel blocks in one single number, we defined p_zero_ as the largest trajectory length percentage p where the effect profile changes its sign from negative to positive. Thus, effect profiles where all ΔΔTr(p)c values are negative were assigned a p_zero_ value of 100%, and effect profiles with all ΔΔTr(p)c values being positive were assigned a p_zero_ value of 1%. The distribution of p_zero_ was approximated using the bootstrap simulated effect profiles.

### Classification of Predominant hERG Channel Blocking Drugs Versus Multichannel Blocking Drugs

We used p_zero_ as separation criterion to classify predominant hERG channel blocking drugs versus multichannel blocking drugs. We determined the classification performance for various threshold values in terms of sensitivity and specificity, using 1000 bootstrap samples of p_zero_ per drug. The thresholds were selected based on visual inspection of the distribution.

## Results

### Heart Rate Correction for Tr(p)

**FIGURE 2:**
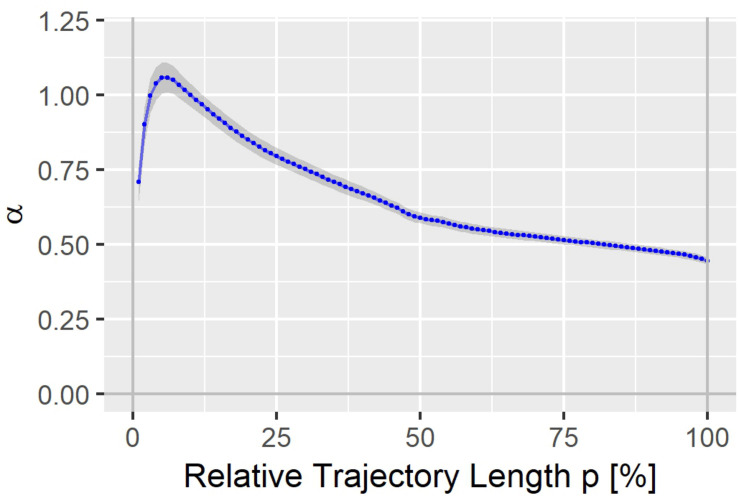
Heart rate correction exponent α for the T vector trajectory quantiles Tr(p). The gray band denotes α ± standard error.

The heart rate correction exponent α for the 100% trajectory quantile Tr(100) was estimated as 0.4437 (*SE*: 0.0119). α increased continuously with decreasing p, reaching a maximum of 1.0597 (*SE*: 0.0501) for the 6% trajectory quantile Tr(6), see Figure 2. The heart rate correction exponents for the individual percentages p are given in the Supplementary File S1. Note that for Tr(100), α lies between the heart rate correction exponents for the QT interval according to the Bazett formula (exponent = 0.5) and the Fridericia formula (exponent = 1/3).

### T Vector Trajectories

**FIGURE 3:**
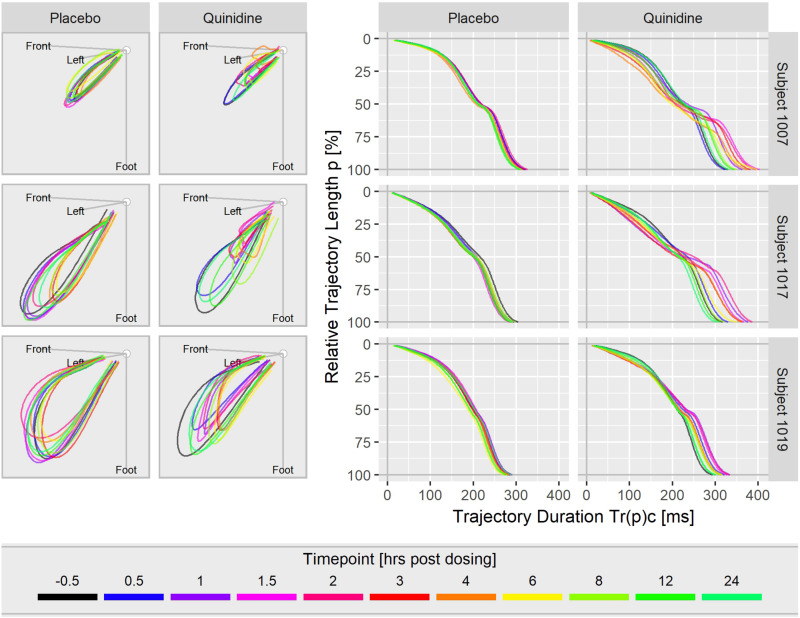
T vector trajectories (left) and heart rate corrected trajectory duration curves (right) from three subjects under placebo and under quinidine treatment during a day. The colored timepoints denote the time in hours post-dosing. Quinidine maximum concentrations were 2130 ng/mL for subject 1007, 1100 ng/mL for subject 1017, 1680 ng/mL for subject 1019.

Figure 3 displays the T vector trajectories and the (heart rate corrected) trajectory duration curves from three subjects in study A during a day under placebo and under quinidine treatment. The T vector trajectories under placebo reflect the diurnal variability within each subject. Following quinidine administration, the T vector trajectories are highly altered, with the manner of change varying between individuals. The T vector duration curves under placebo are well-aligned within the subjects. Following quinidine administration, the trajectory duration curves are strongly affected, such that the early part of the trajectory is passed faster, and the later part is delayed with strongest changes at about 2 h post-dosing.

**FIGURE 4:**
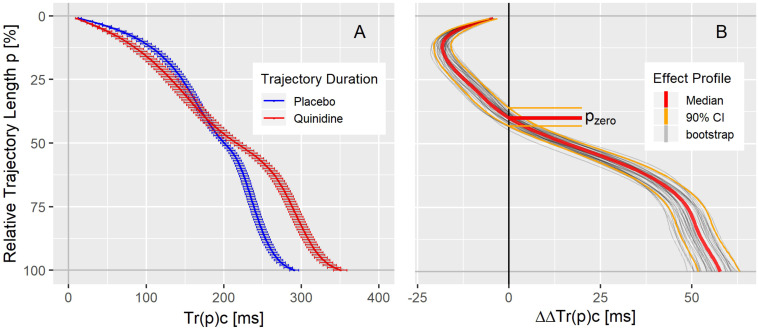
**(A)** Average population T vector trajectory duration curves corrected for heart rate Tr(p)c in study A under placebo (blue) and quinidine (red) calculated for the plasma concentration of 1754 ng/mL with two-sided 90% confidence intervals. **(B)** Quinidine effect profile, showing the placebo corrected change from baseline Tr(p)c. The red lines denote the median, and the orange lines the 90% coverage of the bootstrap simulated continuous effect profiles. Gray lines are a subset of individual bootstrap simulations. The distribution of the zero-transition percentage p_zero_ of the simulated effect profiles (median: 5 and 95% quantile) is indicated by the horizontal lines.

The population average trajectory duration curves for placebo and quinidine at 1754 ng/mL plasma concentration are displayed in panel A of Figure 4, revealing an acceleration effect of quinidine in the initial 40% of the trajectory length and an average delay of about 50 ms at the end of repolarization. The related continuous quinidine effect profile measuring the placebo corrected change from baseline is displayed in panel B of Figure 4. Gray lines represent bootstrap simulated effect profile samples, and the red lines denote the median and the 5 and 95% quantiles of the simulated effect profiles.

As can be seen in the trajectory duration curve for placebo, it takes about 2/3^rd^ of the J-T_end_ interval (Tr(50)c = 200 ms compared to Tr(100)c = 300 ms) to reach 50% of the entire trajectory length. A drug effect profile describes how the drug impacts the times it takes the heart vector to move along the T vector trajectory. Thus, negative ΔΔTr(p)c values (inflected to the left in Figure 4B) indicate accelerated repolarization, positive ΔΔTr(p)c values (inflected to the right in Figure 4B) indicate delayed repolarization. The effect on the 100% trajectory quantile Tr(100)c represents the drug effect on the J-T_end_ interval (corrected for heart rate).

### Drug Effect Profiles in Studies A, B, and C

**FIGURE 5:**
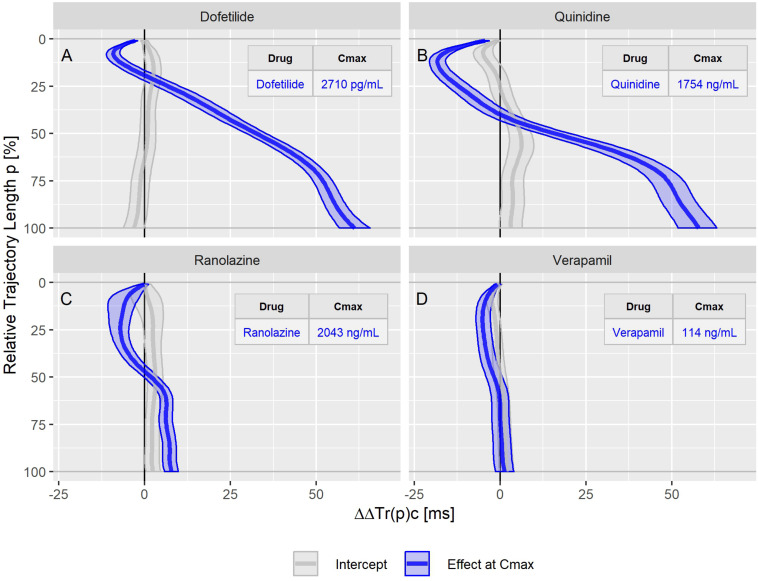
Continuous drug effect profile estimates with 90% confidence bands for the drugs dofetilide **(A)**, quinidine **(B)**, ranolazine **(C)**, and verapamil **(D)** in study A. The blue bands denote the estimated drug effect at the drug’s maximum plasma concentration (Cmax). The gray bands are the model prediction at zero drug concentration.

**FIGURE 6:**
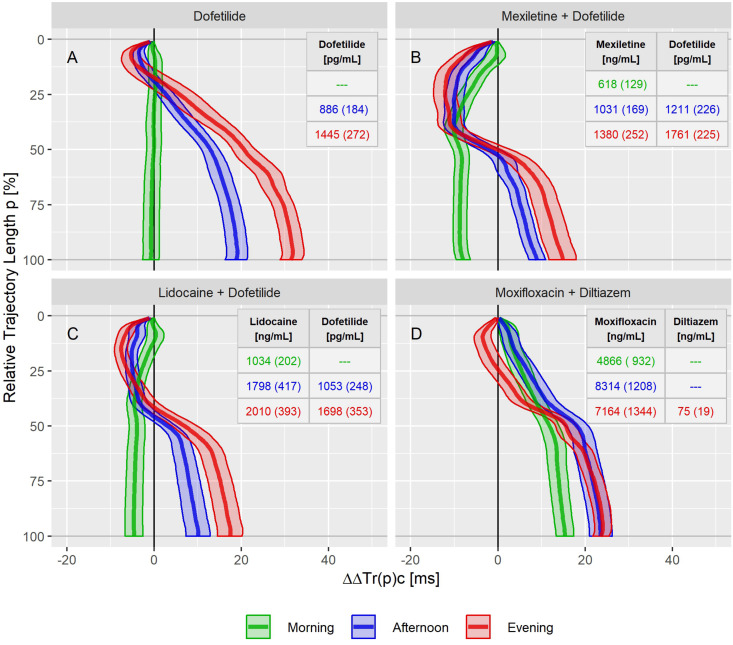
Continuous drug effect profile estimates with 90% confidence bands for dofetilide **(A)**, mexiletine plus dofetilide **(B)**, lidocaine plus dofetilide **(C)**, and moxifloxacin plus diltiazem **(D)** at the three treatment timepoints in study B. Numbers denote the drug’s average (standard deviation) plasma concentration within the timepoints.

**FIGURE 7:**
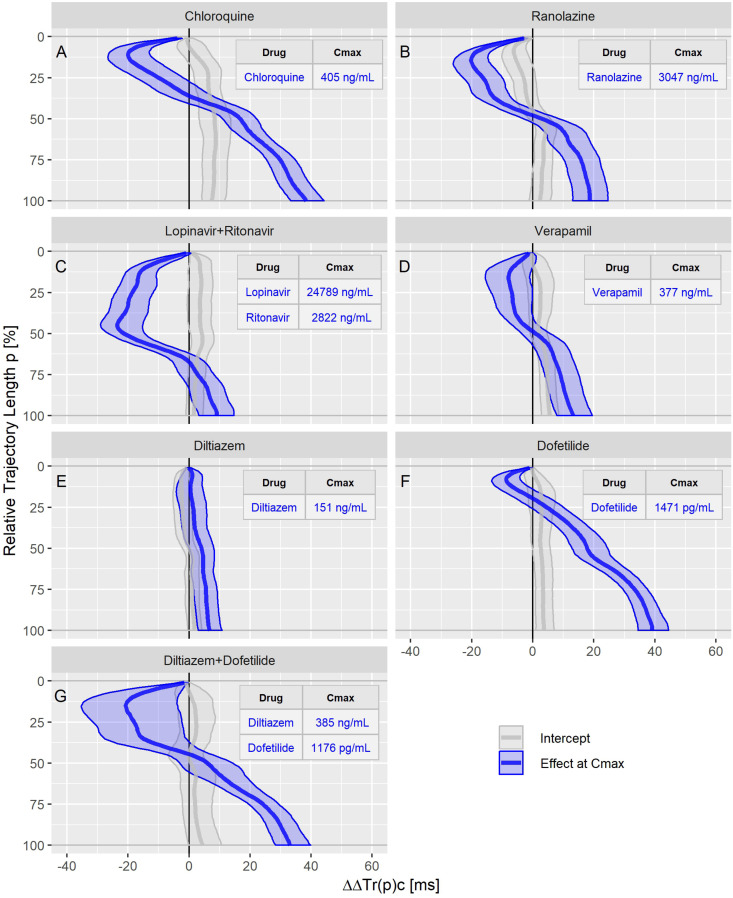
Continuous drug effect profile estimates with 90% confidence bands for chloroquine **(A)**, ranolazine **(B)**, lopinavir plus ritonavir **(C)**, verapamil **(D)**, diltiazem **(E)**, dofetilide **(F)**, and diltiazem plus dofetilide **(G)** in study C. The blue bands denote the estimated drug effect for the drug’s maximum plasma concentration (Cmax). The gray bands are the model prediction at zero drug concentration.

The drug effect profiles for the various treatment conditions in the three studies are displayed in the Figures 5–7. For studies A and C, the effect profiles show the predicted drug effects at the corresponding Cmax drug concentrations, with Cmax as the geometric mean of the subject’s maximum drug concentrations. Furthermore, the prediction of the exposure response models at zero concentration (the so-called intercept) is displayed. An intercept significantly different from zero may indicate violation of the assumption about linear dependency of the drug effect on the drug concentration. For study B, the effect profiles show the drug effects at the average drug concentrations during the three treatment periods (morning, afternoon and evening). Details about the drug concentrations are further described in the appendices.

The dofetilide effect profile could be evaluated under four different conditions: in study A (Figure 5A), study B at afternoon and at evening (Figure 6A), and in study C (Figure 7F). All dofetilide effect profiles show up with similar shapes, characterized by an accelerated repolarization in the initial 20% of the T vector trajectory length and a continuously increased repolarization delay, with a final delay being roughly proportional to the given drug concentration.

The ranolazine effect profile was determined in study A (Figure 5C) and study C (Figure 7B). Both profiles show accelerated repolarization within the initial 50% of the T vector trajectory length, and delay through the end of repolarization. ΔΔTr(100)c was predicted in study A with 2043 ng/mL ranolazine concentration as 7.8 [5.8 to 9.8] ms; in study C with 3047 ng/mL concentration, the effect on ΔΔTr(100)c was 18.7 [13.0 to 24.6] ms.

Quinidine (in study A, Figure 5B) and chloroquine (in study C, Figure 7A) showed pronounced acceleration during the initial T vector trajectory length, and a strong delay through the end of repolarization. The ΔΔTr(100)c effect was 57.8 [51.7 to 63.2] ms for quinidine, and 38.4 [33.3 to 44.3] ms for chloroquine.

An even more pronounced acceleration during the initial 2/3^rd^ of the T vector trajectory length was observed for the drug combination of lopinavir plus ritonavir (Figure 7C) with maximum accelerated repolarization at the 45% trajectory length percentage of ΔΔTr(45)c = −23.5 [−29.0 to −15.2] ms. In the further course of the repolarization, however, there was a delay which ended in a ΔΔTr(100)c value of 9.1 [3.3 to 14.6] ms.

In study A, verapamil caused a minor delay at the end of repolarization with ΔΔTr(100)c = 1.3 [−1.4 to 4.0] ms at a concentration of 114 ng/mL (Figure 5D). In study C, the predicted ΔΔTr(100)c at the concentration of 377 ng/mL was 13.3 [7.9 to 19.5] ms (Figure 7D). The effect profiles for both cases showed a small but clear acceleration during the first T vector trajectory half. The maximum acceleration in study A was observed for ΔΔTr(20)c with −5.1 [−7.1 to −2.9] ms; in study C the maximum acceleration was observed for ΔΔTr(16)c with −8.0 [15.6 to −1.2] ms.

In study B, both mexiletine (Figure 6B) and lidocaine (Figure 6C) shortened the J-T_end_ interval, indicated by the ΔΔTr(100)c value of −8.0 [−9.5 to −6.3] ms for mexiletine at an average concentration of 618 ng/mL and by −4.6 [−6.6 to −2.6] ms for lidocaine at an average concentration of 1034 ng/mL. These acceleration effects were generated within the first 50% of the T vector trajectory length and retained about constant through the end of repolarization. When mexiletine (Figure 6B) and lidocaine (Figure 6C) were combined with dofetilide in the afternoon and evening treatment phases, acceleration persisted during the initial repolarization. From about 40% of the length of the T vector trajectory the repolarization was delayed.

The moxifloxacin effect on repolarization was assessed in the morning and afternoon phases of study B where the effect profiles showed a continuously growing delay without any indication for acceleration (Figure 6D). The additional evening administration of diltiazem resulted in accelerated repolarization during the initial 40% of the T vector trajectory length while leaving the final J-T_end_ prolongation nearly unchanged compared to the afternoon.

Diltiazem was also assessed in study C. Here, the pure diltiazem effect profile showed a slight, continuously increasing delay, ending in a ΔΔTr(100)c value of 6.8 [3.0 to 10.8] ms for the Cmax concentration of 151 ng/mL (Figure 7E). The combination of diltiazem with dofetilide produced an effect profile with pronounced acceleration during the first 40% of the T vector trajectory length and showing large variability in the amount of acceleration (Figure 7G). Notably, the diltiazem Cmax concentrations differed considerably between the eight subjects enrolled in this treatment (from 165 to 1089 ng/mL).

### Classification of Predominant hERG Channel Blocking Drugs Versus Multichannel Blocking Drugs

**FIGURE 8:**
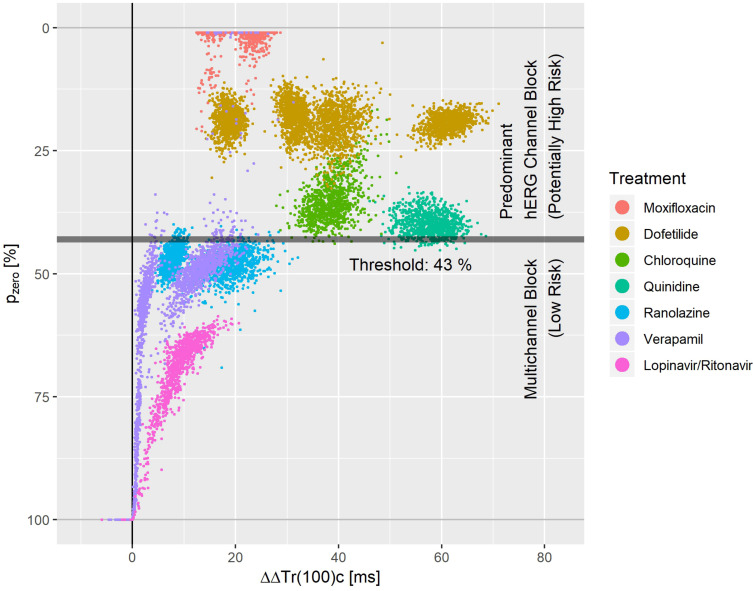
Bootstrap simulated common distribution of p_zero_ (the effect profile’s zero-transition percentage p) and ΔΔTr(100)c (the drug-induced the J-T_end_ prolongation) for predominant hERG channel blocking drugs with potentially higher proarrhythmic risk and multichannel blocking drugs with low proarrhythmic risk.

Moxifloxacin, dofetilide, chloroquine, and quinidine were considered as predominant hERG channel blocking drugs with potentially higher proarrhythmic risk, while ranolazine, verapamil and the combination of lopinavir + ritonavir were considered multichannel blocking drugs with a low proarrhythmic risk (Gintant et al., 2016; Vicente et al., 2019). Mexiletine and lidocaine were not included since they did not prolong the J-T_end_ interval at all. Diltiazem was not included since the observed acceleration tendency during early repolarization when combined with moxifloxacin (Figure 6) or dofetilide (Figure 8) was not visible in the pure diltiazem effect profile (Figure 7). This may indicate limitations of the given model for this compound.

Figure 8 displays the bootstrap simulated common distribution of two repolarization characteristics for the mentioned drugs: the x-axis denotes the drug induced J-T_end_ prolongation measured by ΔΔTr(100)c, and the y-axis denotes the p_zero_ value, that is the percentage of the T vector trajectory length where accelerated repolarization reverts to delayed repolarization. Dofetilide was assessed under four treatment conditions what generated four distinct dofetilide data clouds, reflecting the different drug concentrations. Ranolazine, moxifloxacin, and verapamil were assessed under two treatment conditions each. Thus, two distinct data clouds can be identified for each of these drugs. Notably, the y-levels of the dofetilide, ranolazine, and moxifloxacin data clouds are quite similar for the given drug, indicating independence of p_zero_ from the drug concentrations.

A threshold value of 43% for p_zero_ separated the predominant hERG channel blocking drugs with potentially higher proarrhythmic risk from the multichannel blocking drugs having a low proarrhythmic risk with a sensitivity of 0.99 and a specificity of 0.97.

## Discussion

The T vector reflects the entire myocardial potential distribution at a given moment during repolarization as seen from the body surface ECG. The single-cell myocardial action potential itself is an expression of a complex dynamic process modulated by multiple ion channels and other biological factors (Nerbonne and Kass, 2005). On the scale of the entire heart, the myocardial potential distribution at a certain time during repolarization is generated by myocytes with different action potential characteristics (e.g., sub-endocardial, sub-epicardial, mid-myocardium cells) being activated at different times during the heart’s depolarization process. Acknowledging that the details of the mapping from the collection of single cardiomyocyte action potentials to the whole-organ T vector are beyond our competence, our only assumption is that the temporal progression of the myocardial potential distribution is reflected in the temporal progression of the T vector. Hence, we consider the TVV, the velocity with which the T vector moves along the T vector loop, as an electrocardiographic measure of the repolarization dynamic.

Our results demonstrate that drugs inhibiting myocardial ion channels affect repolarization dynamics in a consistent way which can be quantified by TVV analysis. The signatures extracted from the TVV drug effect profiles allow an estimation of how strongly depolarizing and repolarizing ion currents are blocked by a drug. And finally, they allow highly accurate separation of drugs with potentially higher proarrhythmic risk from drugs with low proarrhythmic risk in the data sets available for the current study.

### Ion Channel Blocks as Reflected in the Continuous Effect Profile

In this study, we describe ventricular repolarization dynamics in terms of the T vector trajectory duration curve, and we introduce continuous drug effect profiles as a new method for measuring drug effects on the repolarization dynamics throughout the entire ventricular repolarization phase as seen in the J-T_end_ interval of the surface ECG.

Figure 3 shows that the T vector trajectories themselves differ very much between subjects and are modulated by cofactors like diurnal variations. Quinidine furthermore affects the trajectory shape and increases trajectory variability. Therefore, it is remarkable that the T vector trajectory duration curves are well-aligned within a given subject under placebo conditions, indicating that the repolarization dynamic follows a subject-specific pattern that is quite constant under normal conditions. For quinidine, we observe a consistent pattern such that the initial 40% of the T vector trajectories are passed faster while the remaining trajectory course is increasingly delayed (Figure 3). This quinidine effect on the cardiac repolarization dynamic in the entire population is quantitatively represented in the continuous effect profile as shown in Figure 4. Our results suggest that the repolarization dynamics and their drug induced changes are less affected by intra and inter-individual confounding factors compared to classical T waveform morphology characteristics. Placing emphasis on the quantification of relative changes in the timing of repolarization appears to contribute to accentuation and more consistent emergence of the drug effect. This held true not only for quinidine but also for all other drugs considered in this study.

This general effect profile pattern, characterized by early acceleration and late delay, was observed for all drugs that block both, the inward late sodium and L-type calcium currents, and the outward potassium currents (quinidine, ranolazine, chloroquine, lopinavir+ritonavir, verapamil). Interestingly, dofetilide as a predominant hERG blocker, also showed a slight but consistent and pronounced acceleration during the initial 20% of the trajectory length (see Figures 5A, 6A, 7F), whereas the effect profile of moxifloxacin showed delayed repolarization along the entire trajectory in both investigated conditions (Figure 6D). One explanation for this observed acceleration could be that although *in vitro* experiments report only a very minor blocking of inward currents by dofetilide (Crumb et al., 2016), TVV analysis is based on *in vivo* measurements, which may also reveal drug effects that reflect more complex ion flow interactions.

Drugs predominantly blocking the late sodium channel (mexiletine – Figure 6B, lidocaine – Figure 6C) showed effect profiles reflecting continuous acceleration during the initial 40% of the trajectories. In the following trajectory section, the accumulated level of acceleration is merely maintained. Furthermore, combining mexiletine or lidocaine with dofetilide (Figures 6B,C) showed a slightly intensified acceleration during the initial trajectory, which then was compensated by the increasing delay along the further trajectory caused by dofetilide’s potassium channel block.

Diltiazem, a calcium antagonist, administered together with moxifloxacin (Figure 6D) shifted the pure moxifloxacin effect profile to the left in the initial half of the trajectory, indicating an acceleration effect by diltiazem. Coadministration of diltiazem plus dofetilide (Figure 7G) created a similar acceleration pattern. However, pure diltiazem did generate a slight but continuously increasing delay over the entire trajectory (Figure 7E). Since the effective diltiazem concentration under single drug condition was between the diltiazem concentrations under the combination with moxifloxacin, respectively dofetilide, the diltiazem concentration cannot explain this somehow contradicting effect.

Our results strongly suggest that the observed acceleration of repolarization reflects a blocking of depolarizing inward ion currents (positive inward charge transfer), while the delay of repolarization reflects a blocking of repolarizing currents (outward transfer of positive charge). This interpretation is supported by the timing of activation of the L-type calcium and the late sodium currents early during ventricular repolarization, compared with the peak of the hERG/IKr current occurring during terminal repolarization of the action potential (Nerbonne and Kass, 2005). We assume that the physiologic activation sequence of the ion channels is preserved by the mapping of the cardiac action potentials onto the ECG. Hence, the observation that accelerations emerged at the lower percentages of the trajectory length (Figures 5–7) is in accordance with the expectation from physiology. Likewise, it fits in the picture that if such acceleration is counteracted by concurrent block of the hERG/IKr-channel, the related reduction of acceleration – and eventually reversion to a delay – becomes particularly evident around the mid-range trajectory length percentages between *p* = 40% and *p* = 60%. Here, we generally observed the strongest accumulation rate of delay for multichannel blocking drugs or drug combinations (Figures 5–7). Note that this corresponds to the region around the peak of the T-wave as discussed in Section “Relation of TVV to QT and J-Tpeak.”

We think that the suggested TVV analysis approach provides a new perspective on repolarization dynamics, linking body surface measurements to cellular electrophysiological effects. We observe that effect profiles from different compounds generally differ in shape and magnitude, while effect profiles of the same compound exhibit stable morphologies over studies (with magnitude scaling related to differences in plasma concentration). Likewise, effect profiles of different drugs inhibiting the same channels to a comparable extent show similar signatures in their effect profiles. This makes us optimistic that the suggested drug effect profiles may turn out in the future to represent a characteristic fingerprint of a drug’s specific ion channel blocking properties and/or extent of proarrhythmic risk. They may even contain more interpretable information about the ion channels involved. Instead of presenting additional details that could possibly support this idea, but would still remain speculative, we would like to offer collaboration to scientific groups specialized in modeling electrophysiology at different scales from the ion channel level over the cellular to the organ and ECG level to verify the TVV observations using existing models.

### TVV and Risk Assessment

We measured the level of balance between blockage of depolarizing and repolarizing ion flow by the p_zero_ parameter, that is the T vector trajectory length percentage where acceleration transitions to delay. A threshold value of p_zero_ = 43% separated predominant hERG channel blocking drugs with potentially higher proarrhythmic risk (moxifloxacin, dofetilide, quinidine, chloroquine) from multichannel blocking drugs with low proarrhythmic risk (ranolazine, verapamil, lopinavir+ritonavir) with sensitivity 0.99 and specificity of 0.97. This is superior to the separation performance reported for J-T_peak_c (Vicente et al., 2016) or for the 40% T vector trajectory duration quantile Tr40c (Bystricky et al., 2019). Tr40c describes the drug-induced change of time to reach 40% of the T vector trajectory length. In contrast, the parameter p_zero_ presented here describes the length fraction of the trajectory where the repolarization process is accelerated. Both parameters are correlated, but in our opinion the new parameter p_zero_ better quantifies the balance of multichannel blocks.

We would like to point out that both mexiletine and lidocaine would be correctly identified as ‘low risk’ compounds using p_zero_ = 43% as threshold. They were omitted from Figure 8, since both drugs had negative ΔΔTr(100)c values with p_zero_ = 100%.

Multiple clusters from the same drug in Figure 8 arise from differences in plasma concentration. For most of the drugs with sufficiently pronounced (>10 ms) prolongation of J-T_end_, the distribution of p_zero_ was almost independent from the J-T_end_ prolongation. This shows that p_zero_ provides substantial information about a possible proarrhythmic risk, which goes beyond QTc.

For a multi-channel blocking drug, the ratio of the strengths of the blockade can change with the drug concentration. Hence, we would expect that p_zero_ may also vary, at least for extreme concentrations. We should therefore assume that more complex separation rules are required when considering additional drugs and extended concentration ranges. Moreover, there is no reason to suppose that lower values of p_zero_ would necessarily correspond to higher proarrhythmic risk. The lower values of p_zero_ observed for moxifloxacin in Figure 8 compared, for example, to dofetilide do not mean that moxifloxacin would indicate a higher propensity for arrhythmia than dofetilide. Rather, there may exist ranges of p_zero_ that reflect potentially critical electrophysiological lability. Again, only the analysis of more compounds will help to shed light on this question.

Finally, the proarrhythmic risk of a drug may depend on factors specific to the individual. A risk assessment is therefore not always conclusive and can be controversial, as with moxifloxacin (Liu et al., 2017; Goto et al., 2020; Yang et al., 2020).

### Relation of TVV to QT and J-T_peak_

Our TVV-based analysis approach targets the J-T_end_ interval, excluding the QRS complex, i.e., the heart’s depolarization process. Therefore, the measured drug-induced change of QTc should be equal to the measured change of Tr(100)c for drugs that do not affect depolarization (the used heart rate correction methods may cause some slight differences between the measurements). For drugs that prolong depolarization, such as chloroquine (Vicente et al., 2019), the observed changes in Tr(100)c will be accordingly smaller than the observed changes in QTc.

The J-T_peak_ interval was corrected for heart rate in the referenced studies A, B and C, similar to the way we corrected heart rate for the trajectory quantiles Tr(p) in our analyses. The heart rate correction exponent for J-T_peak_ was 0.58 (Johannesen et al., 2014), which is similar to the heart rate correction exponent that we identify for the 50 to 55% trajectory length percentages (Figure 2).

The T_peak_ was typically observed to be between 50 and 60% of the T vector trajectory length (Bystricky et al., 2019). This means that the T vector trajectory path up to the point with largest distance from the origin is slightly longer than the trajectory path back to depolarization (visible in Figure 1). Thus, the observed drug effects reflected in the J-T_peak_c parameter (the main parameter of interest in the studies A, B, and C) might be compared with the drug effect profile sections between 50 and 60% of the trajectory length. In study C, named CiPA Phase I ECG Biomarker Validation Study, the prespecified primary endpoint was to show that balanced ion channel-blocking drugs that prolong QTcF would not prolong J-T_end_c defined by an upper bound of the two-sided 90% confidence interval of ΔΔJ-T_end_c < 10 ms at C_max_ (Vicente et al., 2019). This primary endpoint was met for verapamil and lopinavir/ritonavir but was slightly missed by ranolazine (the upper bound of the 90% confidence interval of ΔΔJ-T_peak_c for ranolazine was 12.0 ms). As can be seen in Figure 7, the upper bound of the two-sided 90% confidence band of ranolazine’s effect profile passes the 10 ms at about 50% of the trajectory length. Also, study C reported a negative slope for ΔΔJ-T_peak_c in the exposure-response analysis of lopinavir/ritonavir. This corresponds to the negative effect profile at p between 50 and 60% for lopinavir+ritonavir in Figure 7. Finally, the informative value of the measured ΔΔJ-T_peak_c change for chloroquine was put into question by the authors of study C due to a positive intercept observed in the exposure-response model for chloroquine. This type of non-proportionality was also observed in our analysis, visible in the intercept band for chloroquine in Figure 7, being significantly larger than zero from about *p* = 20 to 100%.

These observations indicate consistency between the TVV analysis and the J-T_peak_c analyses observed in studies A, B, and C. However, we think that the major information characterizing balanced ion channel-blocking drugs is represented over the entire repolarization phase captured in the effect profile, but which is missed to some extent by looking at only one point in time (T_peak_). From a method perspective, providing the effect profile in a continuous fashion constitutes a substantial extension to our previous work (Bystricky et al., 2019). The latter described the effect profile using 10 separate mixed-effects models each associated with a fixed relative trajectory position. Thus, the repolarization period was discretized at 10 equidistant trajectory length percentages. In contrast, the approach presented here rests on a continuous approximation of the effect profile using a number of B-splines. A single mixed-effects model depending on the parameters of this approximation permits estimation of the entire effect profile. This way, the number of free parameters is independent of the sampling density of the effect profile, and dependencies between the trajectory percentages are inherently considered. We are confident that further advantages of this continuous assessment will be demonstrable with more data covering additional compounds becoming available in the future.

### Limitations

Overreading of the ECG annotations was done in an unblinded fashion, but all attempts were made to prevent bias related to drug or drug concentration.

Typically, the applicability of regression models should be evaluated by thoroughly examining the properties of the estimated model parameters and the residuals (Garnett et al., 2018). The functional extension of the mixed effects models used in our analyses adds an additional layer of complexity to the models, making model verification challenging. We therefore regard the proposed functional regression analysis approach, which to our knowledge has been described for the first time in the context of ECG modeling, as a method that requires further scrutiny.

The performance of p_zero_ in separating proarrhythmic drugs from drugs with low proarrhythmic risk was evaluated based on a retrospective analysis of a limited number of drugs used in the three CiPA studies. It will be necessary to study other drugs with known proarrhythmic risk to get an understanding of the generalizability of this method and to propose standard, quantifiable, and reproducible thresholds of concern across a larger and more diverse input dataset.

In addition, it will be necessary to evaluate drugs that specifically affect depolarization to learn how this may influence the drug’s TVV effect profile and p_zero_.

The TVV analysis presented here explores repolarization on a global beat level, not taking into account beat-to-beat variability of repolarization. Acknowledging that beat-to-beat dynamic aspects play a crucial role in the pathway to arrhythmia, extending the TVV analysis for capturing beat-to-beat dynamics may possibly improve proarrhythmic risk assessment given that data of sufficient length is available.

## Conclusion

The TVV-based analysis approach provides new and detailed insights into the repolarization dynamics measured clinically. It links ECG characteristics to cellular electrophysiological effects of multiple ion currents that define repolarization. We have demonstrated that the TVV-derived drug effect profile reveals important information about a drug’s proarrhythmic risk.

## Data Availability Statement

Publicly available datasets were analyzed in this study which can be found here: https://www.physionet.org/content/ecgrdvq/1.0.0/, https://www.physionet.org/content/ecgdmmld/1.0.0/, and https://www.physionet.org/content/ecgcipa/1.0.0/.

## Ethics Statement

The studies involving human participants were reviewed and approved by US Food and Drug Administration (FDA) Research Involving Human Subjects Committee and the local institutional review board. All participants gave written informed consent.

## Author Contributions

WB and CM developed the TVV analysis method. WB analyzed the data. DC supervised this study. All authors contributed to the discussion and prepared the manuscript.

## Conflict of Interest

GG, DB, and DC were employed by AbbVie. WB and CM were working as consultant for AbbVie.

## References

[B1] Acar B. Yi G. Hnatkova K. Malik M. (1999). Spatial, temporal and wavefront direction characteristics of 12-lead T-wave morphology. *Med. Biol. Eng. Comput.* 37 574–584. 10.1007/BF02513351 10723894

[B2] Bates D. Alday P. Kleinschmidt D. Calderón J. B. S. Noack A. Kelman T. (2020). *JuliaStats/MixedModels.jl: v*2.3.0.

[B3] Baumert M. Porta A. Vos M. A. Malik M. Couderc J.-P. Laguna P. (2016). QT interval variability in body surface ECG: measurement, physiological basis, and clinical value: position statement and consensus guidance endorsed by the European Heart Rhythm Association jointly with the ESC Working Group on Cardiac Cellular Electrophysiology. *Europace* 18 925–944. 10.1093/europace/euv405 26823389 PMC4905605

[B4] Berger R. D. Kasper E. K. Baughman K. L. Marban E. Calkins H. Tomaselli G. F. (1997). Beat-to-beat QT interval variability: novel evidence for repolarization lability in ischemic and nonischemic dilated cardiomyopathy. *Circulation* 96 1557–1565. 10.1161/01.cir.96.5.1557 9315547

[B5] Bezanson J. Karpinski S. Viral B. Shah V. B. (2020). *The Julia Programming Language: A Fresh Approach to Technical Computing.* Available online at: https://julialang.org (accessed March 20, 2020).

[B6] Brennan T. P. Tarassenko L. (2012). Review of T-wave morphology-based biomarkers of ventricular repolarisation using the surface electrocardiogram. *Biomed. Signal Process. Control* 7 278–284. 10.1016/j.bspc.2011.05.010

[B7] Britton O. J. Abi-Gerges N. Page G. Ghetti A. Miller P. E. Rodriguez B. (2017). Quantitative comparison of effects of dofetilide, sotalol, quinidine, and verapamil between human ex vivo trabeculae and in silico ventricular models incorporating inter-individual action potential variability. *Front. Physiol.* 8:597. 10.3389/fphys.2017.00597 28868038 PMC5563361

[B8] Bystricky W. Maier C. Gintant G. Bergau D. Kamradt K. Welsh P. (2019). T vector velocity: a new ECG biomarker for identifying drug effects on cardiac ventricular repolarization. *PLoS One* 14:e0204712. 10.1371/journal.pone.0204712 31283756 PMC6613676

[B9] Cai C. Guo P. Zhou Y. Zhou J. Wang Q. Zhang F. (2019). Deep learning-based prediction of drug-induced cardiotoxicity. *J. Chem. Inform. Model.* 59 1073–1084. 10.1021/acs.jcim.8b00769 30715873 PMC6489130

[B10] CiPA (2020). *The CiPA Project Homepage.* Available online at: https://cipaproject.org/about-cipa/ (accessed March 20, 2020).

[B11] Couderc J.-P. (2009). Measurement and regulation of cardiac ventricular repolarization: from the QT interval to repolarization morphology. *Philos. Trans. Ser. A Math. Phys. Eng. Sci.* 367 1283–1299. 10.1098/rsta.2008.0284 19324709 PMC2635501

[B12] Crumb W. J. Vicente J. Johannesen L. Strauss D. G. (2016). An evaluation of 30 clinical drugs against the comprehensive in vitro proarrhythmia assay (CiPA) proposed ion channel panel. *J. Pharmacol. Toxicol. Methods* 81 251–262. 10.1016/j.vascn.2016.03.009 27060526

[B13] Dower G. E. Machado H. B. Osborne J. A. (1980). On deriving the electrocardiogram from vectoradiographic leads. *Clinical Cardiology* 3 87–95. 10.1002/clc.1980.3.2.87 6993081

[B14] Dutta S. Chang K. C. Beattie K. A. Sheng J. Tran P. N. Wu W. W. (2017). Optimization of an in silico cardiac cell model for proarrhythmia risk assessment. *Front. Physiol.* 8:616. 10.3389/fphys.2017.00616 28878692 PMC5572155

[B15] Fossa A. A. (2017). Beat-to-beat ECG restitution: a review and proposal for a new biomarker to assess cardiac stress and ventricular tachyarrhythmia vulnerability. *Ann. Noninvas. Electrocardiol.* 22:e12460. 10.1111/anec.12460 28497858 PMC6931610

[B16] Fossa A. A. Zhou M. (2010). Assessing QT prolongation and electrocardiography restitution using a beat-to-beat method. *Cardiol. J.* 17 230–243. 20535712

[B17] Garnett C. Bonate P. L. Dang Q. Ferber G. Huang D. Liu J. (2018). Scientific white paper on concentration-QTc modeling. *J. Pharmacokinet. Pharmacodyn.* 45 383–397. 10.1007/s10928-017-9558-5 29209907

[B18] Gintant G. Sager P. T. Stockbridge N. (2016). Evolution of strategies to improve preclinical cardiac safety testing. *Nat. Rev. Drug Discov.* 15 457–471. 10.1038/nrd.2015.34 26893184

[B19] Goldberger A. L. Amaral L. A. Glass L. Hausdorff J. M. Ivanov P. C. Mark R. G. (2000). PhysioBank, PhysioToolkit, and PhysioNet: components of a new research resource for complex physiologic signals. *Circulation* 101 E215–E220. 10851218 10.1161/01.cir.101.23.e215

[B20] Goto A. Sakamoto K. Hagiwara-Nagasawa M. Kambayashi R. Chiba K. Nunoi Y. (2020). In vivo analysis of the effects of intravenously as well as orally administered moxifloxacin on the pharmacokinetic and electrocardiographic variables along with its torsadogenic action in the chronic atrioventricular block cynomolgus monkeys. *J. Pharmacol. Sci.* 143 272–280. 10.1016/j.jphs.2020.05.006 32499095

[B21] Graff C. Matz J. Christensen E. B. Andersen M. P. Kanters J. K. Toft E. (2009). Quantitative analysis of T-wave morphology increases confidence in drug-induced cardiac repolarization abnormalities: evidence from the investigational IKr inhibitor Lu 35-138. *J. Clin. Pharmacol.* 49 1331–1342. 10.1177/0091270009344853 19843657

[B22] Han S. Han S. Kim K.-S. Lee H.-A. Yim D.-S. (2019). Usefulness of Bnet, a simple linear metric in discerning Torsades De pointes risks in 28 CiPA drugs. *Front. Pharmacol.* 10:1419. 10.3389/fphar.2019.01419 31849669 PMC6889857

[B23] Haverkamp W. Breithardt G. Camm A. J. Janse M. J. Rosen M. R. Antzelevitch C. (2000). The potential for QT prolongation and pro-arrhythmia by non-anti-arrhythmic drugs: clinical and regulatory implications. Report on a Policy Conference of the European Society of Cardiology. *Cardiovasc. Res.* 47 219–233. 10.1016/s0008-6363(00)00119-x 10947683

[B24] Hnatkova K. Vicente J. Johannesen L. Garnett C. Strauss D. G. Stockbridge N. (2019). Detection of T Wave peak for serial comparisons of JTp interval. *Front. Physiol.* 10:934. 10.3389/fphys.2019.00934 31402872 PMC6670189

[B25] Hondeghem L. M. (2008). QT prolongation is an unreliable predictor of ventricular arrhythmia. *Heart Rhythm.* 5 1210–1212. 10.1016/j.hrthm.2008.05.006 18675236

[B26] Hwang M. Han S. Park M. C. Leem C. H. Shim E. B. Yim D.-S. (2019). Three-dimensional heart model-based screening of proarrhythmic potential by in silico simulation of action potential and electrocardiograms. *Front. Physiol.* 10:1139. 10.3389/fphys.2019.01139 31551815 PMC6738014

[B27] ICH (2005a). *The Clinical Evaluation of QT/QTc Interval Prolongation and Proarrhythmic Potential for Non-Antiarrhythmic Drugs.* Geneva: ICH.

[B28] ICH (2005b). *The non-Clinical Evaluation of the Potential for Delayed Ventricular Repolarization (QT Interval Prolongation) by Human Pharmaceuticals S7B.* Geneva: ICH. 16237859

[B29] Johannesen L. Vicente J. Mason J. W. Erato C. Sanabria C. Waite-Labott K. (2016). Late sodium current block for drug-induced long QT syndrome: results from a prospective clinical trial. *Clin. Pharmacol. Therapeut.* 99 214–223. 10.1002/cpt.205 26259627 PMC5421403

[B30] Johannesen L. Vicente J. Mason J. W. Sanabria C. Waite-Labott K. Hong M. (2014). Differentiating drug-induced multichannel block on the electrocardiogram: randomized study of dofetilide, quinidine, ranolazine, and verapamil. *Clin. Pharmacol. Therapeut.* 96 549–558. 10.1038/clpt.2014.155 25054430

[B31] Li Z. Ridder B. J. Han X. Wu W. W. Sheng J. Tran P. N. (2019). Assessment of an in silico mechanistic model for proarrhythmia risk prediction under the CiPA initiative. *Clin. Pharmacol. Therapeut.* 105 466–475. 10.1002/cpt.1184 30151907 PMC6492074

[B32] Liu X. Ma J. Huang L. Zhu W. Yuan P. Wan R. (2017). Fluoroquinolones increase the risk of serious arrhythmias: a systematic review and meta-analysis. *Medicine* 96:e8273. 10.1097/MD.0000000000008273 29095256 PMC5682775

[B33] Martin R. L. McDermott J. S. Salmen H. J. Palmatier J. Cox B. F. Gintant G. A. (2004). The utility of hERG and repolarization assays in evaluating delayed cardiac repolarization: influence of multi-channel block. *J. Cardiovasc. Pharmacol.* 43 81–90. 10.1080/01926230500431376 15076220

[B34] Mistry H. B. (2018). Complex versus simple models: ion-channel cardiac toxicity prediction. *PeerJ* 6:e4352. 10.7717/peerj.4352 29423349 PMC5804316

[B35] Nerbonne J. M. Kass R. S. (2005). Molecular physiology of cardiac repolarization. *Physiol. Rev.* 85 1205–1253. 10.1152/physrev.00002.2005 16183911

[B36] O’Hara T. Virág L. Varró A. Rudy Y. (2011). Simulation of the undiseased human cardiac ventricular action potential: model formulation and experimental validation. *PLoS Comput. Biol.* 7:e1002061. 10.1371/journal.pcbi.1002061 21637795 PMC3102752

[B37] Okada J.-I. Yoshinaga T. Kurokawa J. Washio T. Furukawa T. Sawada K. (2015). Screening system for drug-induced arrhythmogenic risk combining a patch clamp and heart simulator. *Sci. Adv.* 1:e1400142. 10.1126/sciadv.1400142 26601174 PMC4640654

[B38] Passini E. Britton O. J. Lu H. R. Rohrbacher J. Hermans A. N. Gallacher D. J. (2017). Human in silico drug trials demonstrate higher accuracy than animal models in predicting clinical pro-arrhythmic cardiotoxicity. *Front. Physiol.* 8:668. 10.3389/fphys.2017.00668 28955244 PMC5601077

[B39] RCoreTeam (2020). *R: A Language and Environment for Statistical Computing.* Vienna: R Foundation for Statistical Computing.

[B40] Redfern W. S. Carlsson L. Davis A. S. Lynch W. G. MacKenzie I. Palethorpe S. (2003). Relationships between preclinical cardiac electrophysiology, clinical QT interval prolongation and torsade de pointes for a broad range of drugs: evidence for a provisional safety margin in drug development. *Cardiovasc. Res.* 58 32–45. 10.1016/s0008-6363(02)00846-5 12667944

[B41] Ridder B. J. Leishman D. J. Bridgland-Taylor M. Samieegohar M. Han X. Wu W. W. (2020). A systematic strategy for estimating hERG block potency and its implications in a new cardiac safety paradigm. *Toxicol. Appl. Pharmacol.* 394:114961. 10.1016/j.taap.2020.114961 32209365 PMC7166077

[B42] Roden D. M. (2004). Drug-induced prolongation of the QT interval. *N. Engl. J. Med.* 350 1013–1022. 10.1056/NEJMra032426 14999113

[B43] Sager P. T. Gintant G. Turner J. R. Pettit S. Stockbridge N. (2014). Rechanneling the cardiac proarrhythmia safety paradigm: a meeting report from the Cardiac Safety Research Consortium. *Am. Heart J.* 167 292–300. 10.1016/j.ahj.2013.11.004 24576511

[B44] Sahli-Costabal F. Seo K. Ashley E. Kuhl E. (2020). Classifying drugs by their arrhythmogenic risk using machine learning. *Biophys. J.* 118 1165–1176. 10.1016/j.bpj.2020.01.012 32023435 PMC7063479

[B45] Tomek J. Bueno-Orovio A. Passini E. Zhou X. Minchole A. Britton O. (2019). Development, calibration, and validation of a novel human ventricular myocyte model in health, disease, and drug block. *eLife* 8:e48890. 10.7554/eLife.48890 31868580 PMC6970534

[B46] Verrier R. L. Klingenheben T. Malik M. El-Sherif N. Exner D. V. Hohnloser S. H. (2011). Microvolt T-wave alternans physiological basis, methods of measurement, and clinical utility–consensus guideline by international society for holter and noninvasive electrocardiology. *J. Am. Coll. Cardiol.* 58 1309–1324. 10.1016/j.jacc.2011.06.029 21920259 PMC4111570

[B47] Verrier R. L. Malik M. (2013). Electrophysiology of T-wave alternans: mechanisms and pharmacologic influences. *J. Electrocardiol.* 46 580–584. 10.1016/j.jelectrocard.2013.07.003 23948521

[B48] Vicente J. Johannesen L. Hosseini M. Mason J. W. Sager P. T. Pueyo E. (2016). Electrocardiographic biomarkers for detection of drug-induced late sodium current block. *PLoS One* 11:e0163619. 10.1371/journal.pone.0163619 28036334 PMC5201270

[B49] Vicente J. Johannesen L. Mason J. W. Crumb W. J. Pueyo E. Stockbridge N. (2015). Comprehensive T wave morphology assessment in a randomized clinical study of dofetilide, quinidine, ranolazine, and verapamil. *J. Am. Heart Assoc.* 4:e001615. 10.1161/JAHA.114.001615 25870186 PMC4579946

[B50] Vicente J. Zusterzeel R. Johannesen L. Ochoa-Jimenez R. Mason J. W. Sanabria C. (2019). Assessment of multi-ion channel block in a Phase I randomized study design: results of the CiPA Phase I ECG biomarker validation study. *Clin. Pharmacol. Therapeut.* 105 943–953. 10.1002/cpt.1303 30447156 PMC6654598

[B51] Yang P.-C. DeMarco K. R. Aghasafari P. Jeng M.-T. Dawson J. R. D. Bekker S. (2020). A Computational pipeline to predict cardiotoxicity: from the atom to the rhythm. *Circ. Res.* 126 947–964. 10.1161/CIRCRESAHA.119.316404 32091972 PMC7155920

[B52] Zemzemi N. Bernabeu M. O. Saiz J. Cooper J. Pathmanathan P. Mirams G. R. (2013). Computational assessment of drug-induced effects on the electrocardiogram: from ion channel to body surface potentials. *Br. J. Pharmacol.* 168 718–733. 10.1111/j.1476-5381.2012.02200.x 22946617 PMC3579290

