# Supplementary material for: Identification of Drug-Induced Multichannel Block and Proarrhythmic Risk in Humans Using Continuous T Vector Velocity Effect Profiles Derived From Surface Electrocardiograms
Source: Front Physiol. 2020 Sep 18;11:567383. doi: 10.3389/fphys.2020.567383 (PMC7530300; doi:10.3389/fphys.2020.567383)
Supplement: Supplementary file 1 [file Data_Sheet_1.PDF]

TVV Supplement - Dependency of Tr(p) on RR

Werner Bystricky, AbbVie

2020-May-22

Overview

This document is a supplement of the study:

Identification of drug-induced multichannel block and proarrhythmic risk in humans using continuous T vector velocity effect profiles derived from surface electrocardiograms

It describes the dependency of the T-vector trajectory quantiles $Tr(p)$, on the heart rate, respectively the RR interval, where $p \in 1, \ldots, 100$ denotes the percentage of the total T vector trajectory length.

Data

The data are from the three CiPA studies A, B, and C, published at PhysioNet.

Only placebo or zero-drug ECGs were used.

Number of subjects: 104

Number of ECGs: 3590

Methods

The TVV is measured by the time required to reach p% of the total T vector trajectory length, here denoted as Tr_p.

Assuming a power law $Tr_p(RR) = \beta_p * RR^{\alpha_p}$ with RR in seconds, α_p is estimated by fitting mixed effects models (R method *lme4::lmer()*) to the log-transformed data:

$$log(Tr_p(RR)) = log(\beta_p) + \alpha_p * log(RR) + \epsilon$$

with subject as random effect on the slope and intercept.

The estimated heart rate correction exponents α_p were used to calculate the heart rate corrected T vector trajectory quantiles $Tr(p)c = \frac{Tr(p)}{RR^{\alpha_p}}$.

Results

The following figure displays the estimated heart rate correction exponent $\alpha_p \pm SE$ for the individual percentiles $p \in \{1, \ldots, 100\}$.

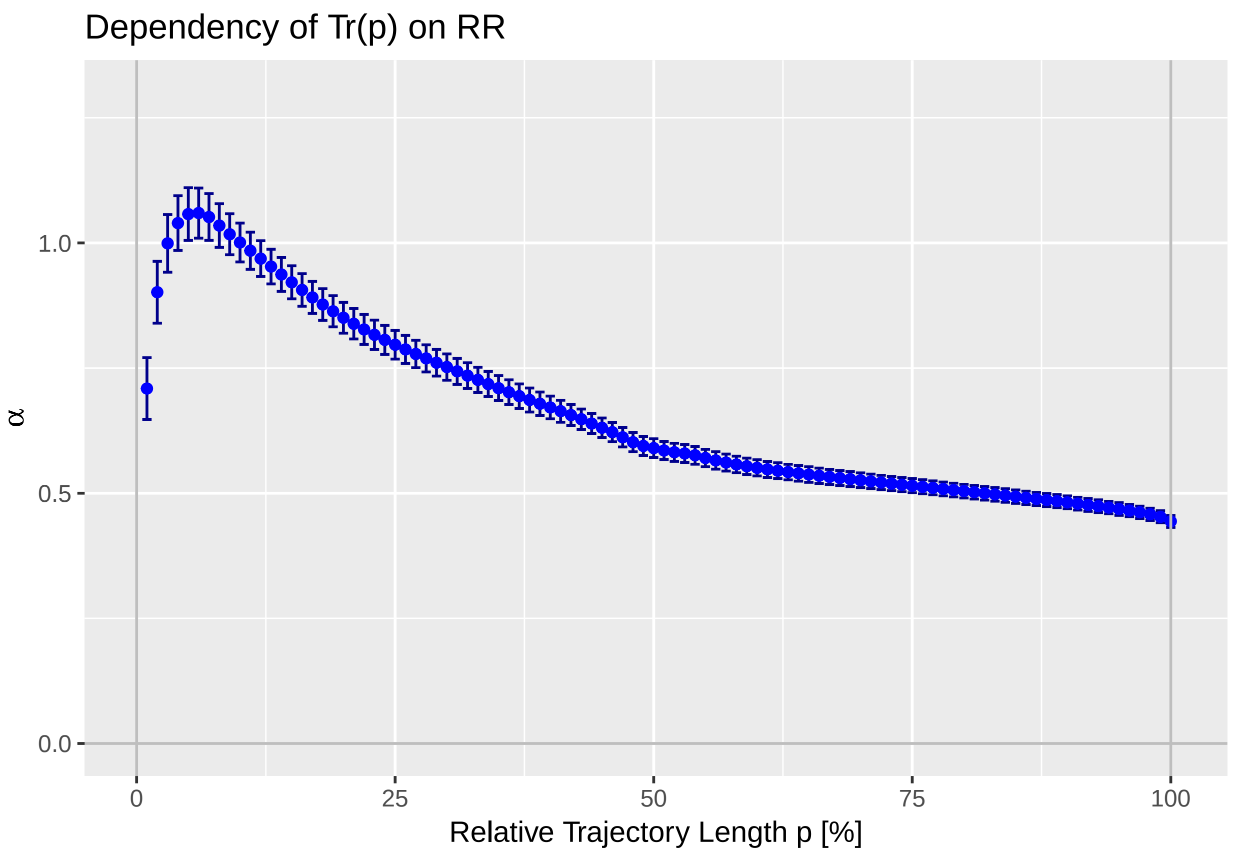

The following table lists for each percentage p the estimated exponent α_p with its standard error (SE):

p	alpha	SE
1	0.7090	0.0615
2	0.9015	0.0617
3	0.9990	0.0575
4	1.0395	0.0547
5	1.0575	0.0527
6	1.0597	0.0501
7	1.0517	0.0467
8	1.0346	0.0436
9	1.0172	0.0410
10	1.0008	0.0389
11	0.9844	0.0372
12	0.9685	0.0358
13	0.9527	0.0347
14	0.9369	0.0338
15	0.9213	0.0330
16	0.9060	0.0324
17	0.8911	0.0320
18	0.8769	0.0315
19	0.8634	0.0311
20	0.8505	0.0307
21	0.8384	0.0303
22	0.8271	0.0299
23	0.8164	0.0294

p	alpha	SE
24	0.8062	0.0290
25	0.7965	0.0285
26	0.7872	0.0280
27	0.7781	0.0276
28	0.7693	0.0271
29	0.7606	0.0266
30	0.7520	0.0262
31	0.7434	0.0259
32	0.7348	0.0256
33	0.7264	0.0254
34	0.7180	0.0252
35	0.7097	0.0249
36	0.7017	0.0247
37	0.6938	0.0244
38	0.6862	0.0240
39	0.6787	0.0235
40	0.6713	0.0228
41	0.6638	0.0219
42	0.6560	0.0211
43	0.6477	0.0204
44	0.6391	0.0199
45	0.6307	0.0195
46	0.6219	0.0193
47	0.6116	0.0193
48	0.6019	0.0192
49	0.5943	0.0189
50	0.5901	0.0185
51	0.5853	0.0183
52	0.5818	0.0181
53	0.5794	0.0179
54	0.5757	0.0178
55	0.5702	0.0176
56	0.5654	0.0173
57	0.5612	0.0170
58	0.5573	0.0167
59	0.5537	0.0164
60	0.5505	0.0162
61	0.5476	0.0160
62	0.5449	0.0158
63	0.5422	0.0157
64	0.5397	0.0156
65	0.5372	0.0154
66	0.5348	0.0153
67	0.5325	0.0152
68	0.5303	0.0150
69	0.5280	0.0149
70	0.5258	0.0147
71	0.5236	0.0146
72	0.5214	0.0145
73	0.5192	0.0144
74	0.5170	0.0143
75	0.5148	0.0142

p	alpha	SE
76	0.5127	0.0141
77	0.5105	0.0140
78	0.5084	0.0139
79	0.5062	0.0138
80	0.5041	0.0137
81	0.5019	0.0136
82	0.4998	0.0136
83	0.4976	0.0135
84	0.4953	0.0134
85	0.4931	0.0133
86	0.4909	0.0133
87	0.4886	0.0132
88	0.4863	0.0131
89	0.4840	0.0130
90	0.4816	0.0129
91	0.4791	0.0129
92	0.4766	0.0128
93	0.4740	0.0127
94	0.4712	0.0126
95	0.4683	0.0125
96	0.4653	0.0124
97	0.4618	0.0123
98	0.4579	0.0122
99	0.4528	0.0121
100	0.4437	0.0119
