# Supplementary material for: Identification of Drug-Induced Multichannel Block and Proarrhythmic Risk in Humans Using Continuous T Vector Velocity Effect Profiles Derived From Surface Electrocardiograms
Source: Front Physiol. 2020 Sep 18;11:567383. doi: 10.3389/fphys.2020.567383 (PMC7530300; doi:10.3389/fphys.2020.567383)
Supplement: Supplementary file 2 [file Data_Sheet_2.PDF]

TVV Supplement - Drug Concentrations

Werner Bystricky, AbbVie

2020-May-22

Overview

This document is a supplement of the study:

Identification of drug-induced multichannel block and proarrhythmic risk in humans using continuous T vector velocity effect profiles derived from surface electrocardiograms

It provides further details about the time course of drug concentrations in the three studies A, B, and C, as given in the published data at PhysioNet.

Study A

Study A assessed the drug effects of dofetilide, quinidine, ranolazine and verapamil on repolarization in a single-dose crossover design on 22 subjects.

For further details of study A see Johannesen2014.

The following graphs display the drug concentration time courses for the individual subjects during the four drug administration periods. The black lines denote the average of the drug concentrations per nominal timepoint post dosing.

Administration: Dofetilide

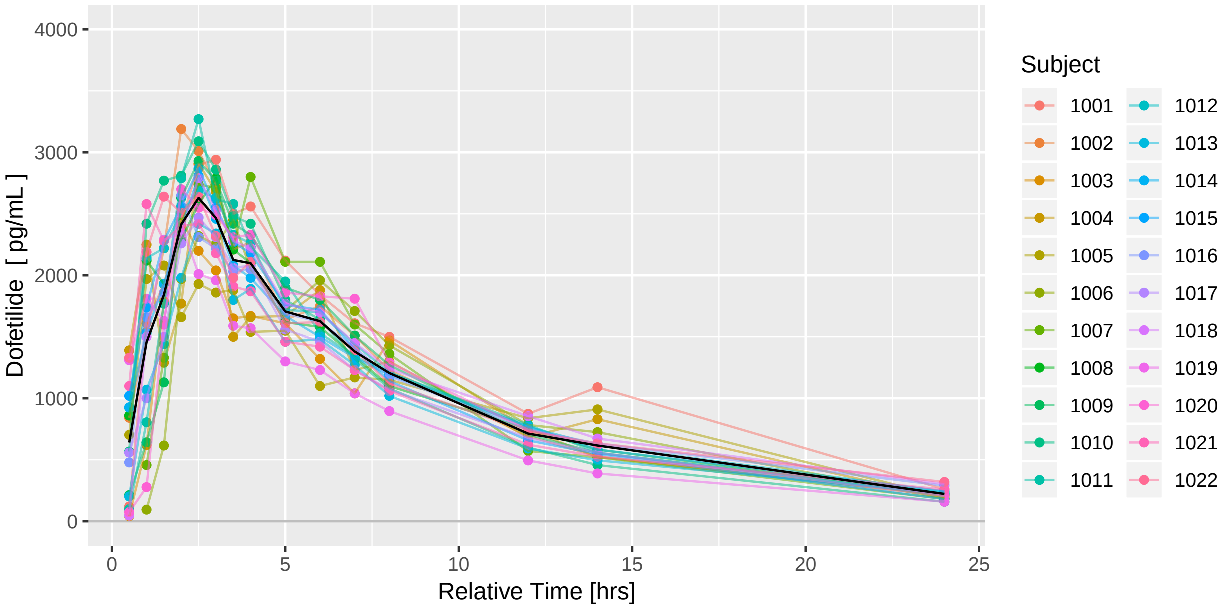

Administration: Quinidine

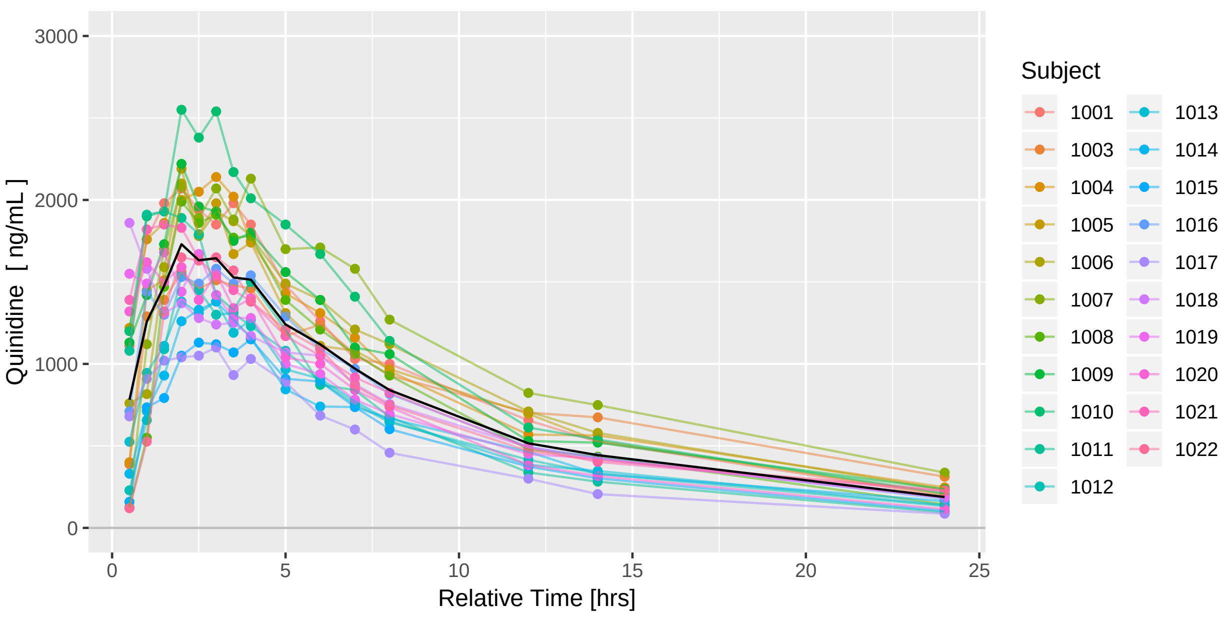

Administration: Ranolazine

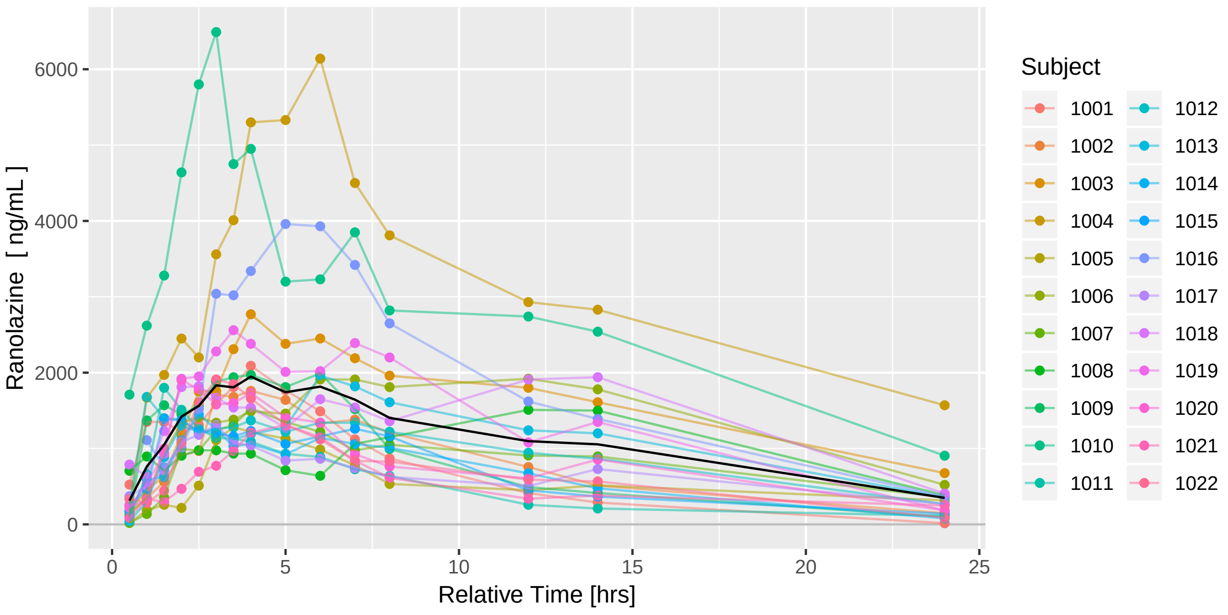

Administration: Verapamil

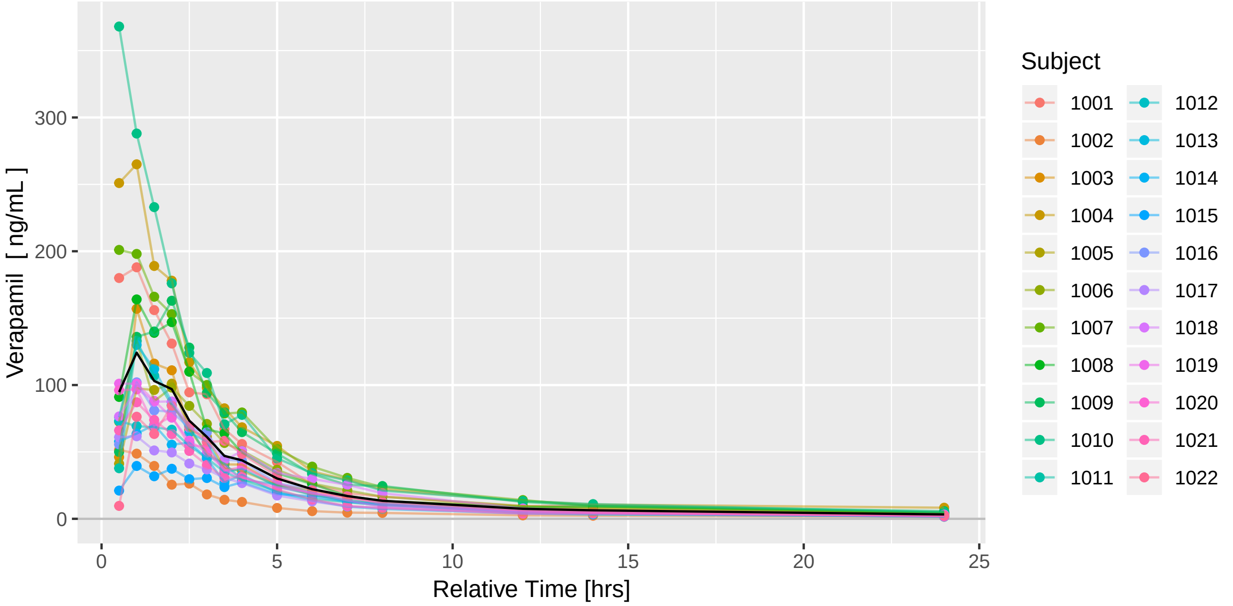

Study B

Study B investigated the following drug and drug combinations in a five-period crossover design:

- P1: Pure Dofetilide (Dof)
- P2: Combination mexiletine (Mex) + dofetilide
- P3: Combination lidocaine (Lid) + dofetilide
- P4: Placebo (Pla)
- P5: Combination moxifloxacin (Mox) + diltiazem (Dil)

The 22 subjects were dosed three times during the day (morning, afternoon, evening) according to the following (simplified) schema:

Administration	Morning	Afternoon	Evening
Dofetilide	—	Dof	Dof
Mexiletine + Dofetilide	Mex	Mex + Dof	Mex + Dof
Lidocaine + Dofetilide	Lid	Lid + Dof	Lid + Dof
Moxifloxacin + Diltiazem	Mox	Mox	Mox + Dil

For further details of study B see Johannesen2016.

The following graphs display the drug concentration time courses for the individual subjects during the various drug administration periods. The black lines denote the average of the drug concentrations per nominal timepoint.

Administration: Dofetilide

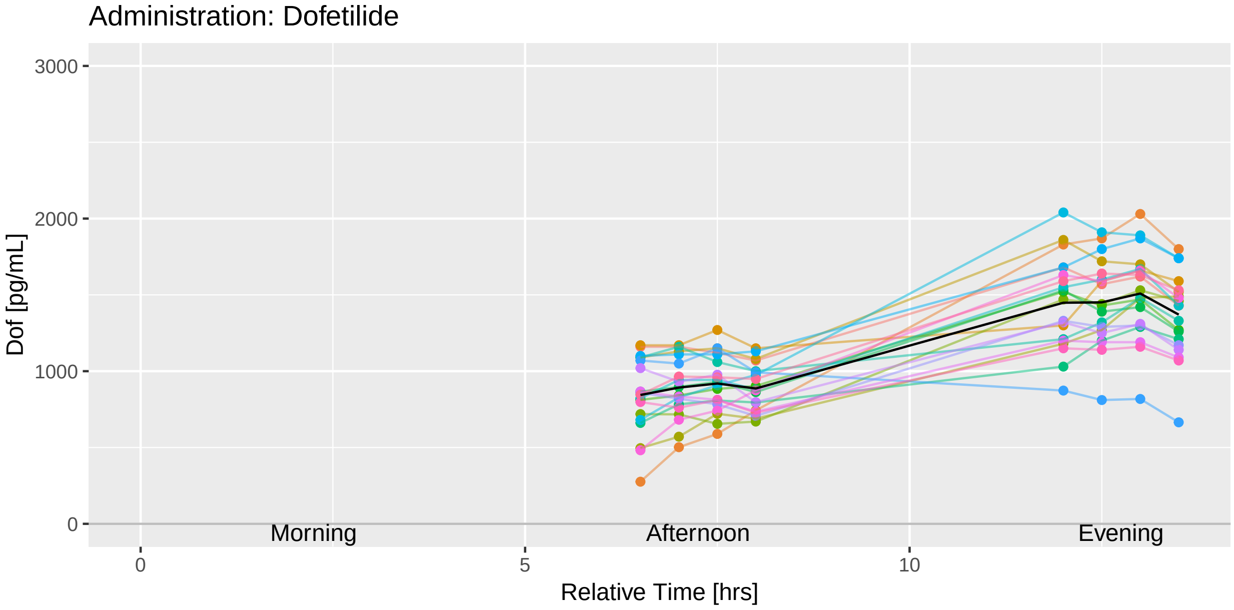

Administration: Mexiletine + Dofetilide

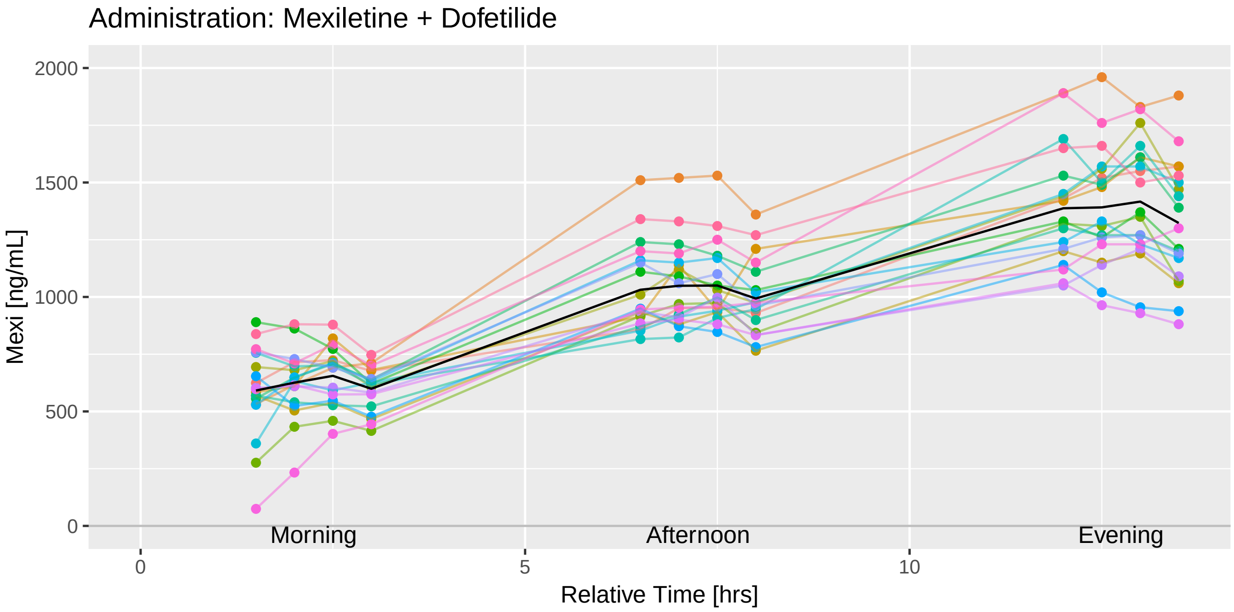

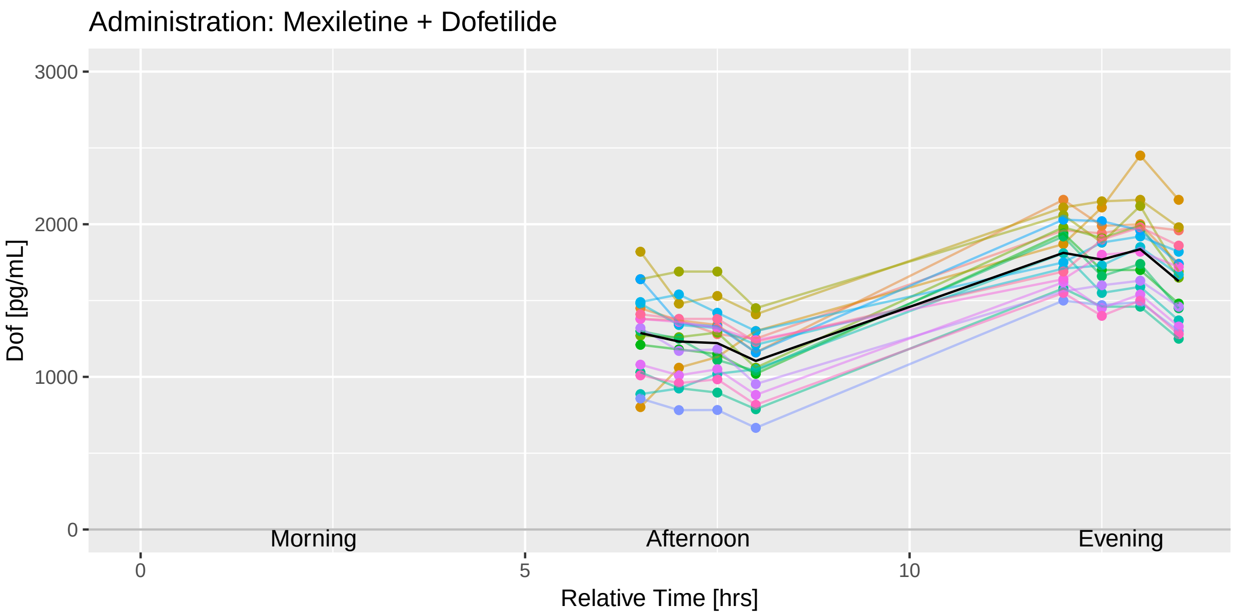

Administration: Lidocaine + Dofetilide

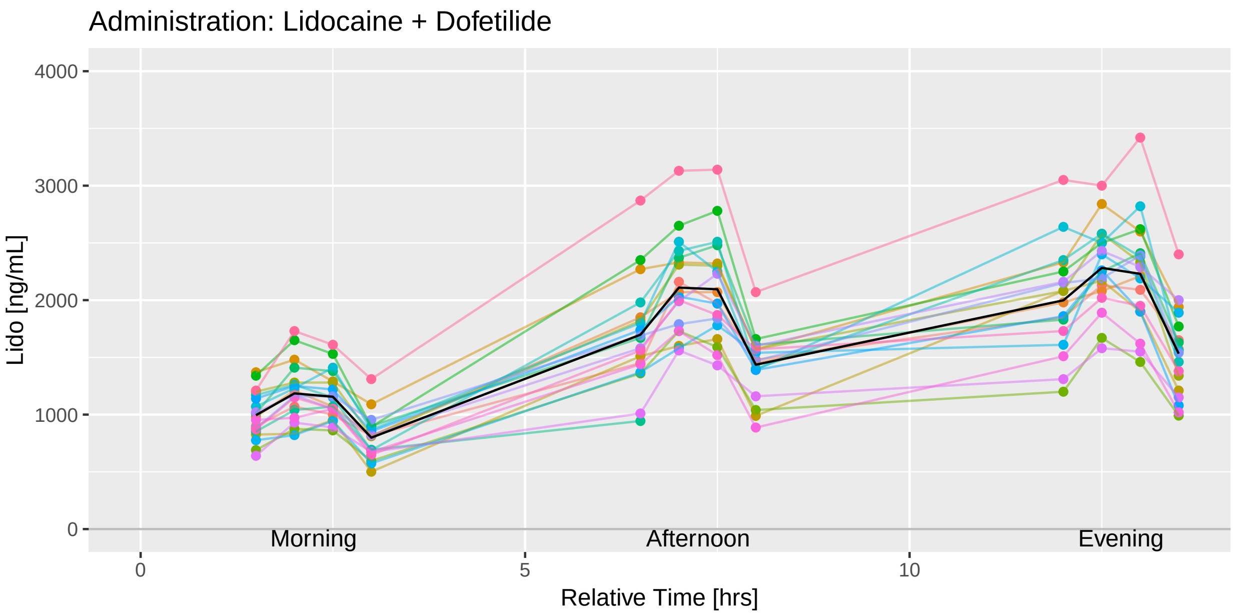

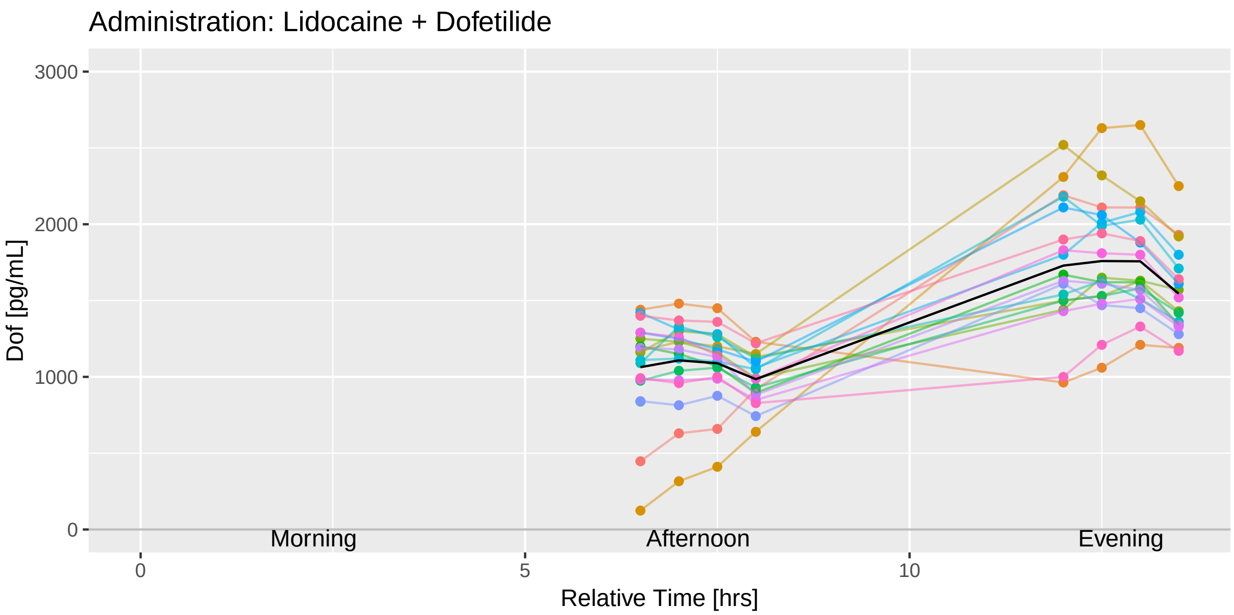

Administration: Moxifloxacin + Diltiazem

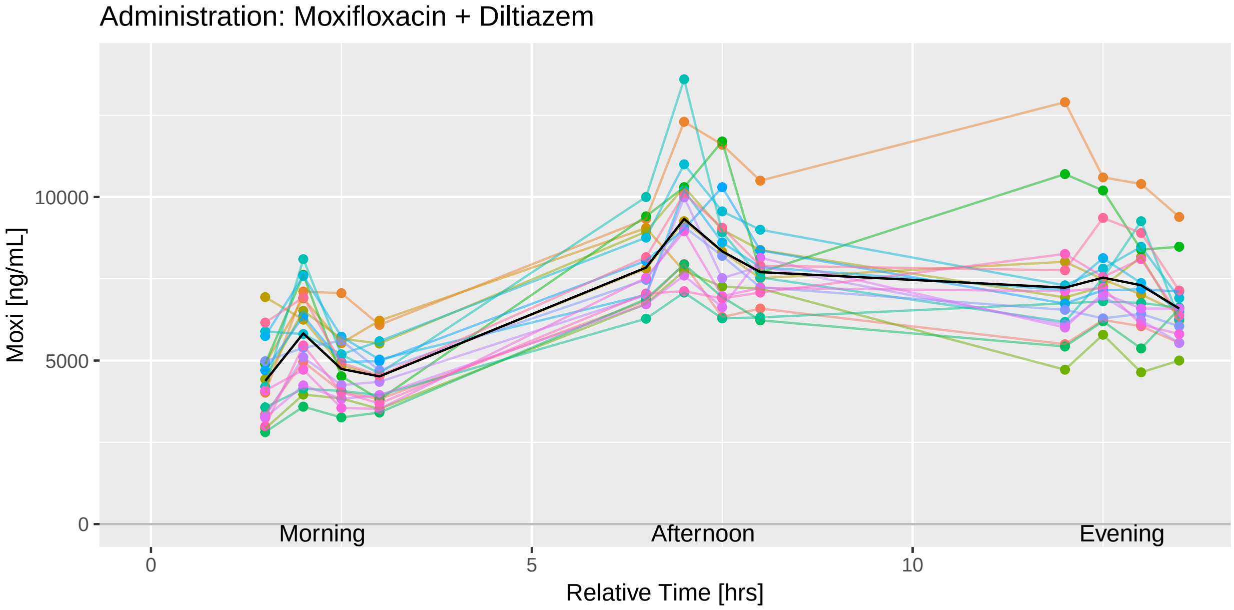

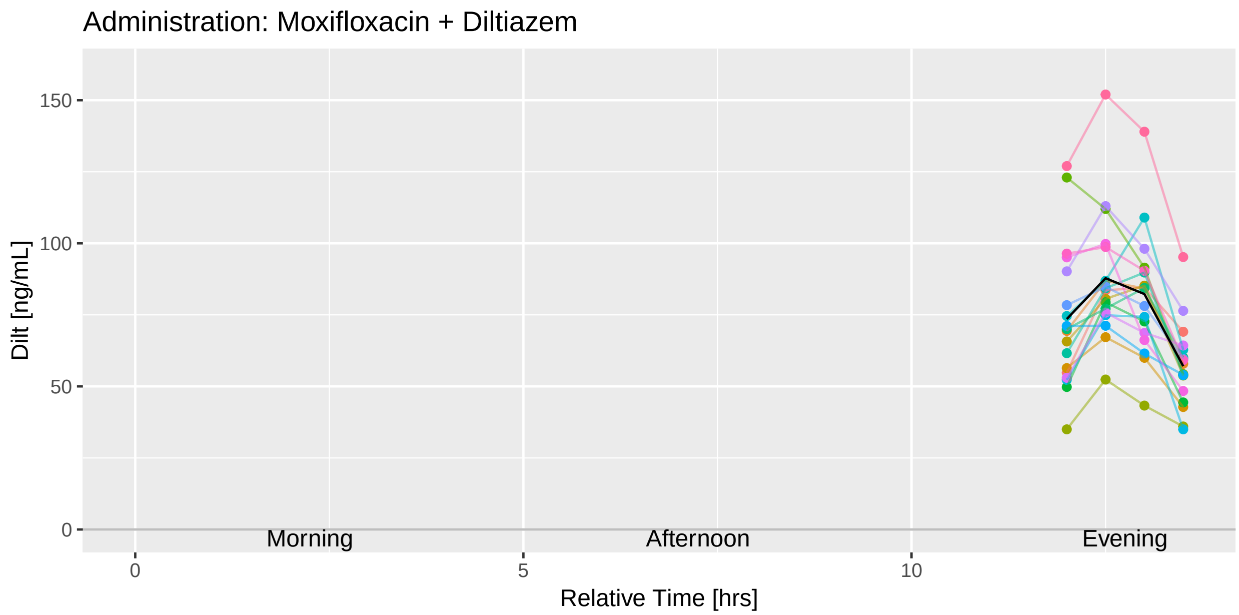

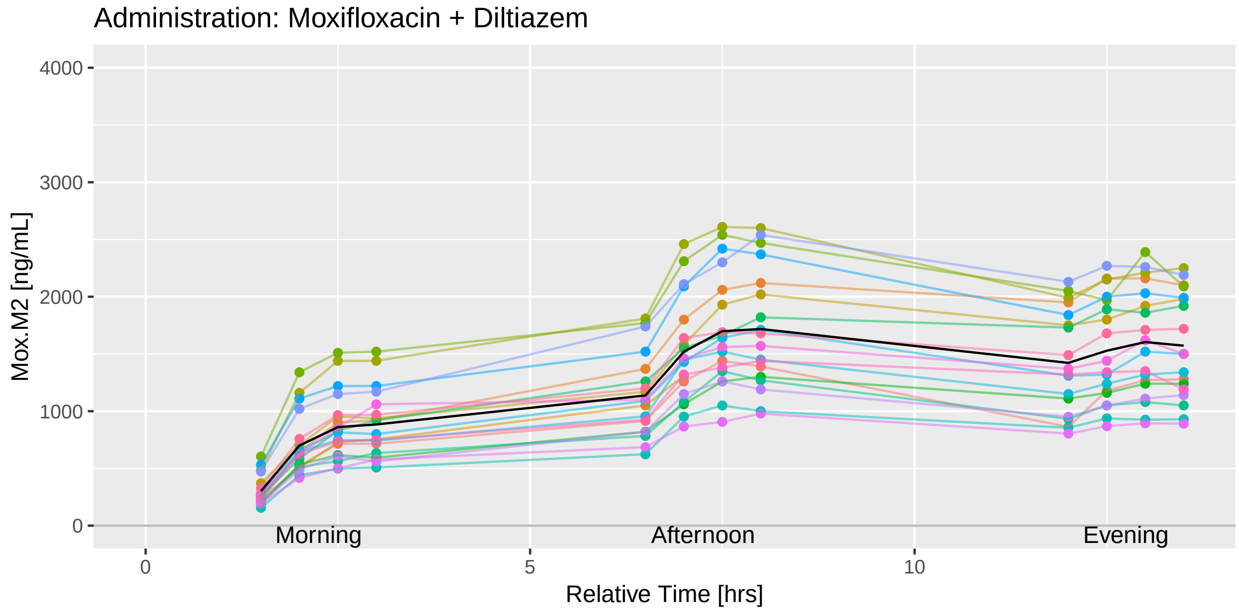

Study C

Study C consisted of two parts:

- Part 1: Placebo-controlled, one-period parallel design to assess the effect of ranolazine, verapamil and the combination of lopinavir+ritonavir on repolarization with a three days drug administration schema.
- Part 2: Crossover design to access the effect of dofetilide and of dofetilide plus diltiazem with a three days drug administration schema applied on 10 subjects.

For further details of study C see Vicente2018.

The following graphs display the drug concentration time courses for the individual subjects during the various drug administration periods. The black lines denote the average of the drug concentrations per

nominal timepoint.

Administration: Chloroquine

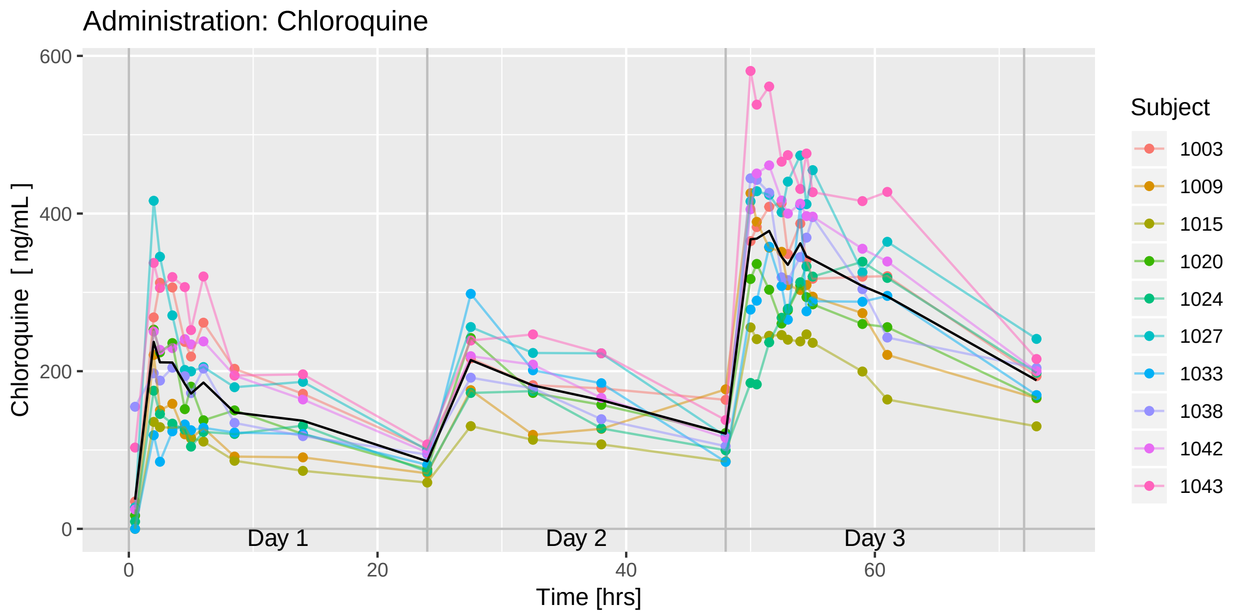

Administration: Ranolazine

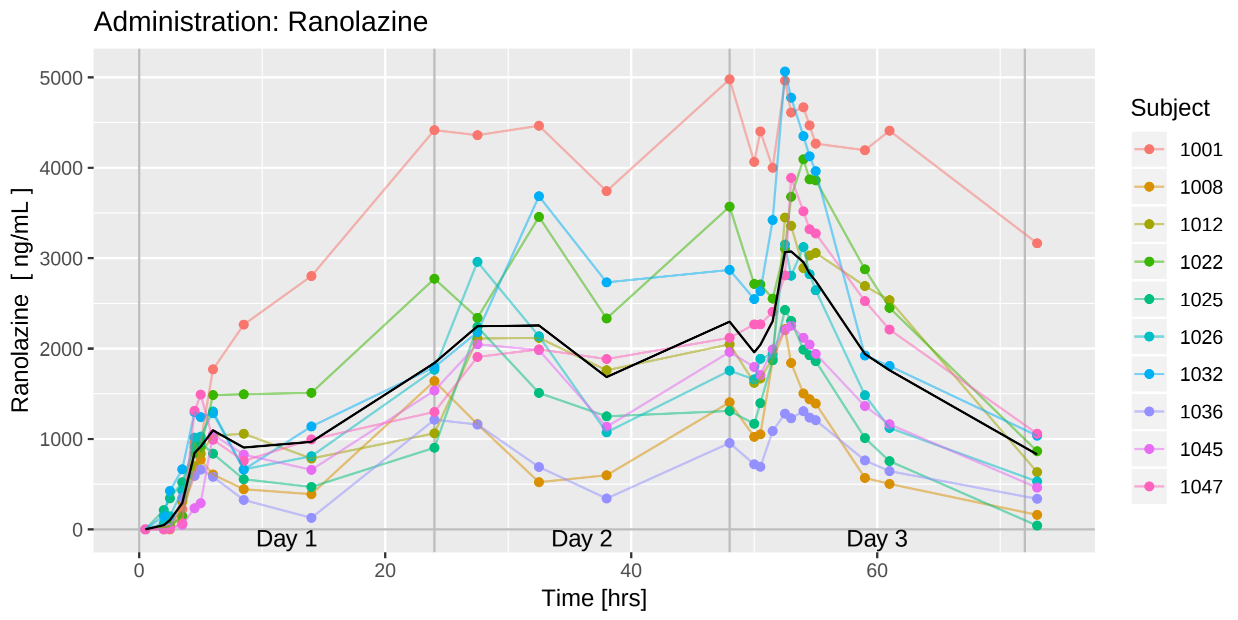

Administration: Verapamil

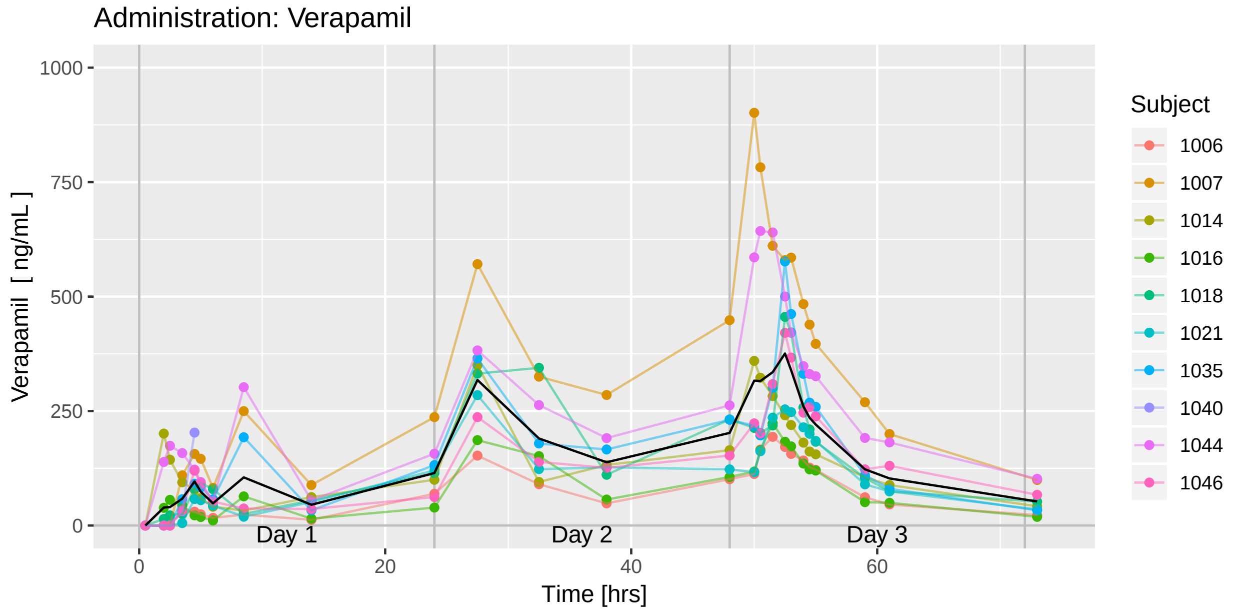

Administration: Lopinavir+Ritonavir

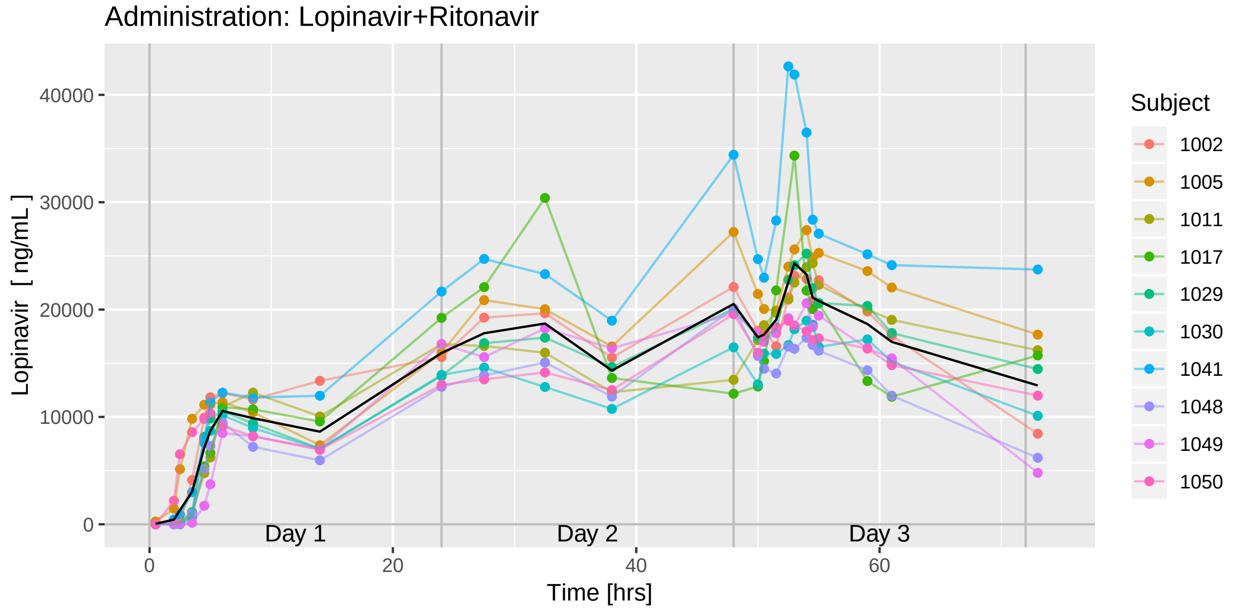

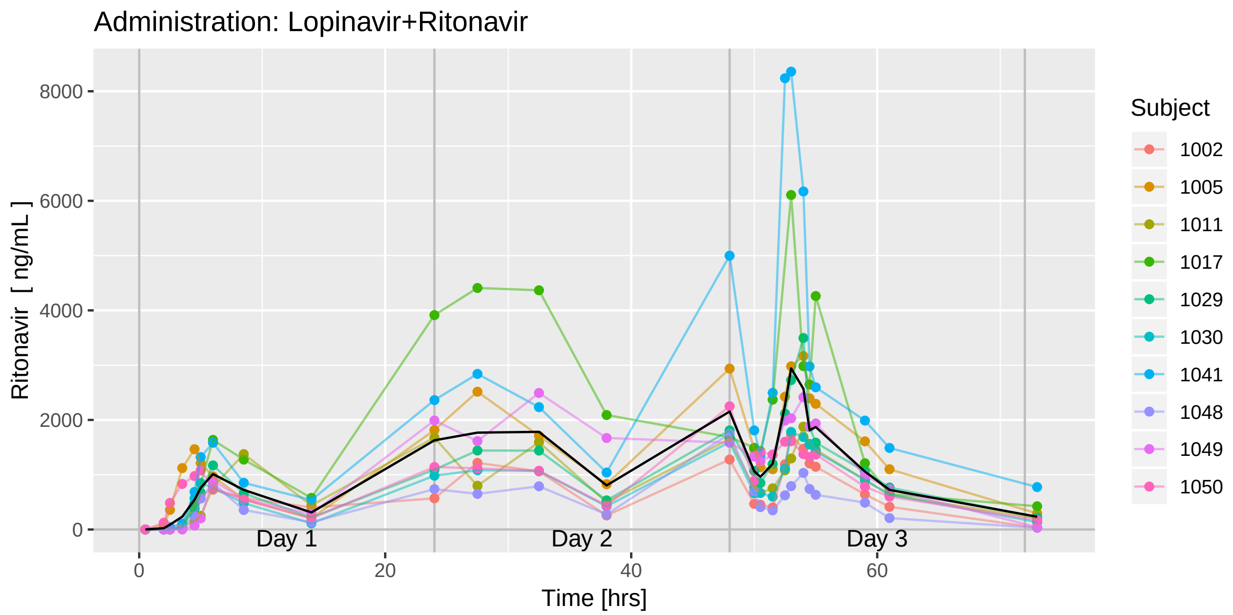

Administration: Diltiazem+Dofetilide

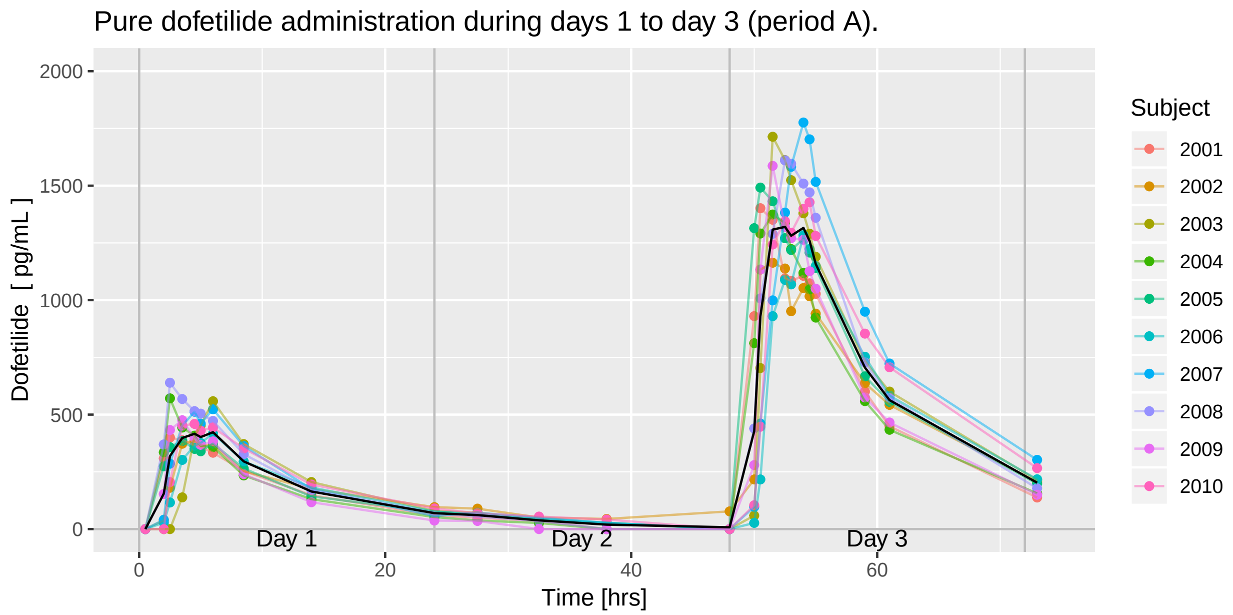

Pure diltiazem administration during day 1 and day 2 (period B).

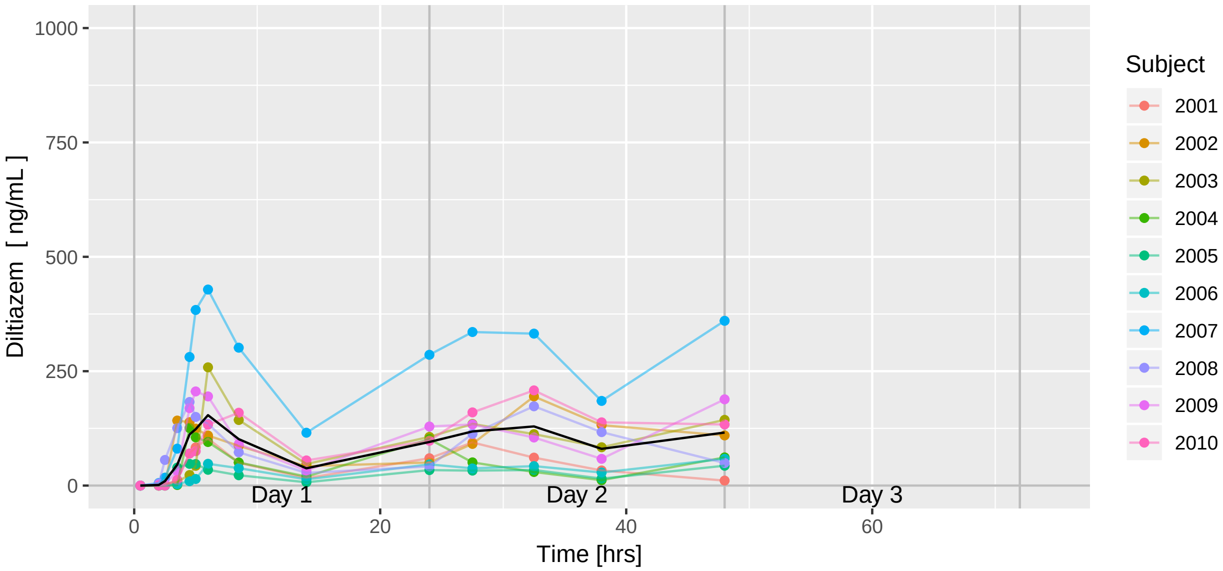

Combined diltiazem + dofetilide administration at day 3 (period B).

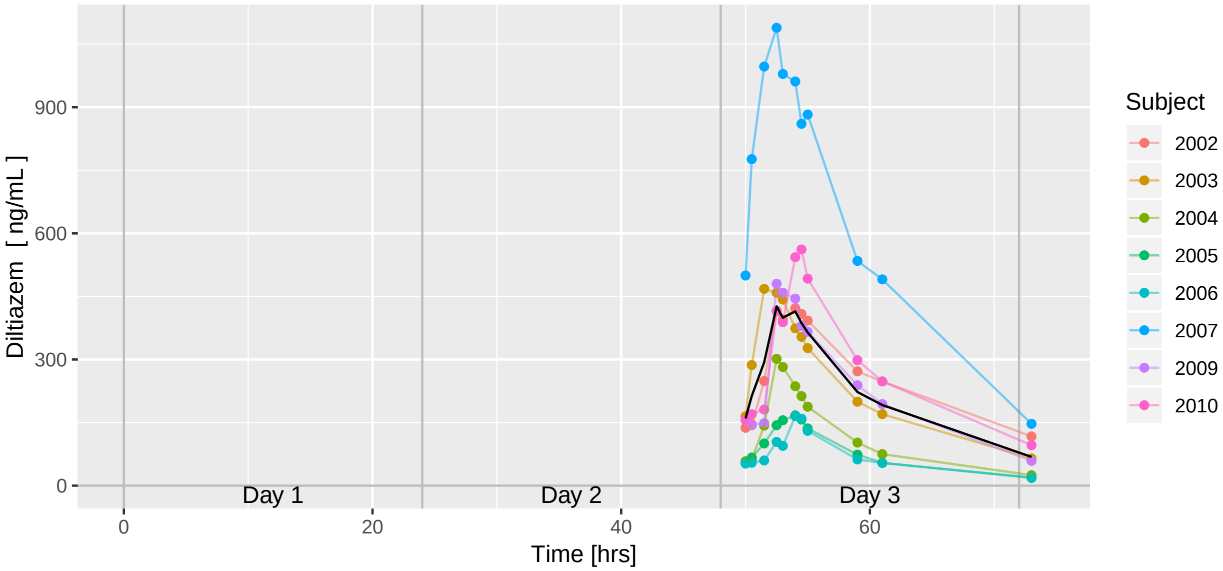

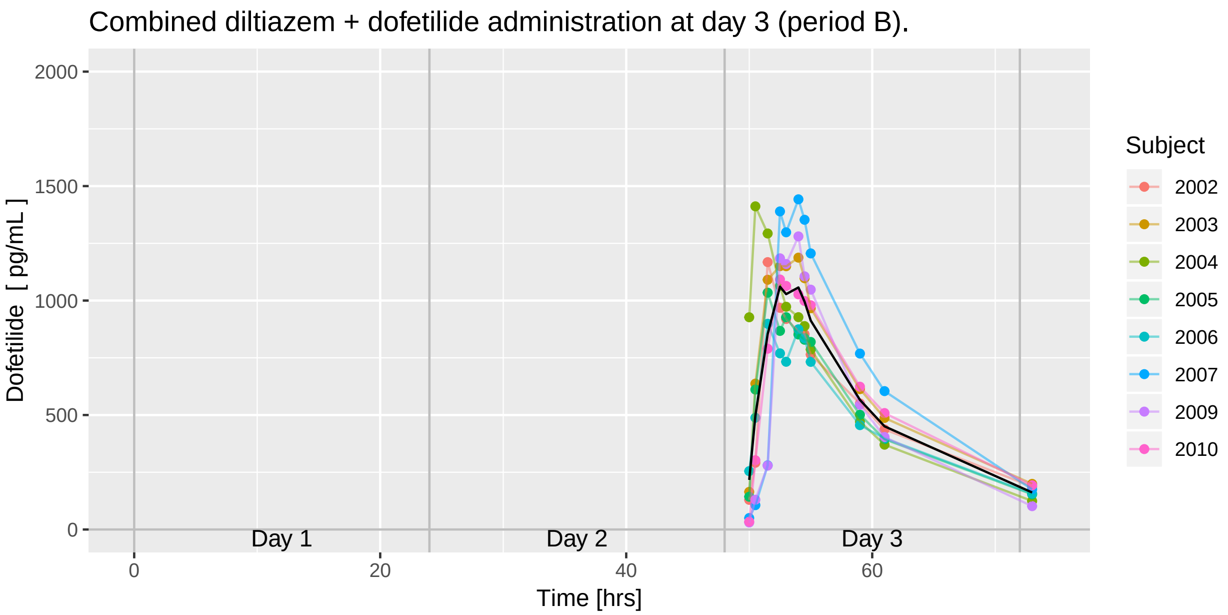

Combined diltiazem + dofetilide administration at day 3 (period B).
Dofetilide [pg/mL]
2000
1500
1000
500
0
Day 1
Day 2
Day 3
0
20
40
60
Time [hrs]
Subject
2002
2003
2004
2005
2006
2007
2009
2010
