# Supplementary material for: Identification of Drug-Induced Multichannel Block and Proarrhythmic Risk in Humans Using Continuous T Vector Velocity Effect Profiles Derived From Surface Electrocardiograms
Source: Front Physiol. 2020 Sep 18;11:567383. doi: 10.3389/fphys.2020.567383 (PMC7530300; doi:10.3389/fphys.2020.567383)
Supplement: Supplementary file 3 [file Data_Sheet_3.PDF]

TVV Supplement - Functional Model

Werner Bystricky, AbbVie

2020-May-22

Overview

This document is a supplement of the study:

Identification of drug-induced multichannel block and proarrhythmic risk in humans using continuous T vector velocity effect profiles derived from surface electrocardiograms

It describes the principal structure of the functional mixed effects models on the example of study A, and presents code for simulating the distribution of the drug effect profiles.

Functional mixed effects model

Let the model

$$\Delta\Delta Tr(p)c_{ik} = (\theta_0(p) + \eta_{0,i}(p)) + (\theta_1(p) + \eta_{1,i}(p)) \times C_{ik}$$

describe the placebo corrected change from baseline drug effect on a specific T vector trajectory quantile $Tr(p)c$ in study A.

To combine the models for the individual perentages $p \in \{1, 2, \ldots, 100\}$, the p-dependent model parameters are parametrized by polynomial splines (R *method splines::bs*) with degree 3, defined by a sequence of knots. The figure below displays the spline basis functions as used for the models in study A.

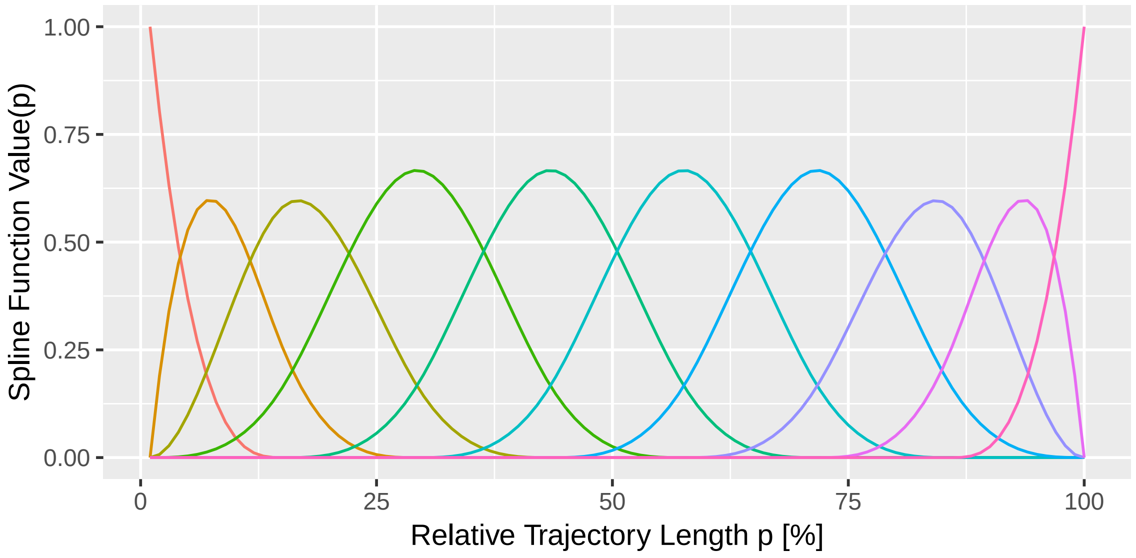

Figure 1: Spline Basis Functions

Here, 8 equally spaced knots between 1 and 100 define K = 10 overlapping spline basis functions $S_k(p),\ k \in \{1, \ldots, K\}$.

To build the design matrix for the functional model, each ECG record ends up in 100 data rows representing the individual p values. Furthermore, each p-dependent model parameter ends up in K = 10 columns representing the linear combinations of the spline basis functions.

To illustrate that, let $\mathbf{y}^{(i)}$ denote the vector of observed placebo corrected change from baseline values of the i-th ECG at plasma concentration $C^{(i)}$ for the T vector trajectory length percentages $p \in \{1, \ldots, 100\}$. Then, the (fixed effects) design matrix $\mathbf{X^{(i)}}$ related to the ECG (i) is

$$\mathbf{X^{(i)}} = \begin{pmatrix} S_1(1) & S_2(1) & \cdots & S_K(1) & S_1(1)C^{(i)} & S_2(1)C^{(i)} & \cdots & S_K(1)C^{(i)} \\ S_1(2) & S_2(2) & \cdots & S_K(2) & S_1(2)C^{(i)} & S_2(2)C^{(i)} & \cdots & S_K(2)C^{(i)} \\ \vdots & \vdots & \ddots & \vdots & \vdots & \vdots & \ddots & \vdots \\ S_1(100) & S_2(100) & \cdots & S_K(100) & S_1(100)C^{(i)} & S_2(100)C^{(i)} & \cdots & S_K(100)C^{(i)} \end{pmatrix}$$

The fixed effects parameter vector of the functional model is

$$\Theta = \begin{pmatrix} \theta_{0,1} & \theta_{0,2} & \cdots & \theta_{0,K} & \theta_{1,1} & \theta_{1,2} & \cdots & \theta_{1,K} \end{pmatrix}^T$$

and the predicted drug effect for all ECGs and percentages p would be calculated as $\hat{\mathbf{y}} = \mathbf{X}\hat{\Theta}$.

Note: In study C with the mixed effects model containing categorial parameters, each categorial level must be parametrized accordingly.

Julia code

The following **Julia** code fits the functional mixed effects model from above, and performs the two-step bootstrap sampling for determining the distribution of the prediced drug effects.

The function **BS(...)** reads the data file containing the data of the design matrix, plus columns for the subject (S), ECG (R), and measured placebo corrected change from baseline (Y). The functional model parameters are denoted as I_Bk := $\theta_{0,k}$ and C_Bk := $\theta_{1,k}$.

For each bootstrap simulation, the data file is resampled on the subject level by method **resample2(...)**, which in turn resamples each subject-related data set on the ECG level by method **resample(...)**. The estimated fixed effects are exported and used for calculating the drug effect profile distribution within the **R** system.

For details about mixed models see Mixed-effects models in Julia.

```
using Tables, MixedModels, DataFrames, DelimitedFiles, CSV, Random, Dates

function resample(df, col)
    items = by(df, Symbol(col), N = Symbol(col) => length)
    number_items = size(items,1)
    items.X = rand(1:number_items,number_items)
    arrM = Array{Union{Nothing,DataFrame}}(nothing,number_items)
    for i in 1:number_items
        item = items[items[i,:X], Symbol(col)]
        arrM[i] = df[df[:,Symbol(col)] .== item,:]
    end
    df1 = vcat(arrM...)
end

function resample2(df, col1, col2)
```

```
    items = by(df, Symbol(col1), N = Symbol(col1) => length)
    number_items = size(items,1)
    items.X = rand(1:number_items,number_items)
    arrM = Array{Union{Nothing,DataFrame}}(nothing,number_items)
    for i in 1:number_items
        item = items[items[i,:X], Symbol(col1)]
        df1 = df[df[:,Symbol(col1)] .== item,:]
        arrM[i] = resample(df1, col2)
    end
    df1 = vcat(arrM...)
end

function BS(drug, source, N)
    data_file = string("FD_", drug, ".txt")
    out_file = string("BSF_", drug, ".txt")
    df = CSV.read(joinpath(source, data_file); types=Dict("S"=>String))

    f = @formula(Y ~ 0 + I_B1 + I_B2 + I_B3 + I_B4 + I_B5 + I_B6 + I_B7 + I_B8 + I_B9 + I_B10
      + C_B1 + C_B2 + C_B3 + C_B4 + C_B5 + C_B6 + C_B7 + C_B8 + C_B9 + C_B10
      + (
        0 + I_B1 + I_B2 + I_B3 + I_B4 + I_B5 + I_B6 + I_B7 + I_B8 + I_B9 + I_B10
        + C_B1 + C_B2 + C_B3 + C_B4 + C_B5 + C_B6 + C_B7 + C_B8 + C_B9 + C_B10 | S)
        )

    for r in 1:N
        df1 = resample2(df, "S", "R")
        fm1 = fit(MixedModel, f, df1)
        fe1 = fixef(fm1,false)
        cols = length(fe1)
        BS = Array{Union{Missing,Float64}}(missing, 1, cols)
        for c in 1:cols
            BS[1,c] = fe1[c]
        end
        CSV.write(joinpath(source, out_file), DataFrame(BS),
          writeheader=false, delim='\t', newline="\r\n", append=true)
    end
end
```

End of Supplement
